# Coral restoration – A systematic review of current methods, successes, failures and future directions

**DOI:** 10.1371/journal.pone.0226631

**Published:** 2020-01-30

**Authors:** Lisa Boström-Einarsson, Russell C. Babcock, Elisa Bayraktarov, Daniela Ceccarelli, Nathan Cook, Sebastian C. A. Ferse, Boze Hancock, Peter Harrison, Margaux Hein, Elizabeth Shaver, Adam Smith, David Suggett, Phoebe J. Stewart-Sinclair, Tali Vardi, Ian M. McLeod

**Affiliations:** 1 TropWATER, James Cook University, Townsville, Qld, Australia; 2 Commonwealth Scientific and Industrial Research Organisation (CSIRO), Brisbane, Qld, Australia; 3 University of Queensland, Brisbane, Qld, Australia; 4 Marine Ecology Consultant, Magnetic Island, Qld, Australia; 5 Reef Ecologic, Townsville, Qld, Australia; 6 Future Earth Coasts, Leibniz Centre for Tropical Marine Research (ZMT), Bremen, Germany; 7 Marine Ecology Department, Faculty of Biology and Chemistry (FB2), University of Bremen, Bremen, Germany; 8 The Nature Conservancy, Arlington, Virginia, United States of America; 9 Southern Cross University, Lismore, NSW, Australia; 10 University of Technology Sydney, Sydney, NSW, Australia; 11 ECS for NOAA Fisheries, Office of Science & Technology, Silver Spring, MD, United States of America; Universita degli Studi di Genova, ITALY

## Abstract

Coral reef ecosystems have suffered an unprecedented loss of habitat-forming hard corals in recent decades. While marine conservation has historically focused on passive habitat protection, demand for and interest in active restoration has been growing in recent decades. However, a disconnect between coral restoration practitioners, coral reef managers and scientists has resulted in a disjointed field where it is difficult to gain an overview of existing knowledge. To address this, we aimed to synthesise the available knowledge in a comprehensive global review of coral restoration methods, incorporating data from the peer-reviewed scientific literature, complemented with grey literature and through a survey of coral restoration practitioners. We found that coral restoration case studies are dominated by short-term projects, with 60% of all projects reporting less than 18 months of monitoring of the restored sites. Similarly, most projects are relatively small in spatial scale, with a median size of restored area of 100 m^2^. A diverse range of species are represented in the dataset, with 229 different species from 72 coral genera. Overall, coral restoration projects focused primarily on fast-growing branching corals (59% of studies), and report survival between 60 and 70%. To date, the relatively young field of coral restoration has been plagued by similar ‘growing pains’ as ecological restoration in other ecosystems. These include 1) a lack of clear and achievable objectives, 2) a lack of appropriate and standardised monitoring and reporting and, 3) poorly designed projects in relation to stated objectives. Mitigating these will be crucial to successfully scale up projects, and to retain public trust in restoration as a tool for resilience based management. Finally, while it is clear that practitioners have developed effective methods to successfully grow corals at small scales, it is critical not to view restoration as a replacement for meaningful action on climate change.

## Introduction

Coral reef ecosystems have suffered an unprecedented loss of habitat-forming hard corals in the past few decades [1–5]. Reefs are subject to a suite of chronic and acute anthropogenic disturbances including declining water quality, destructive fishing practices, over-harvesting of reef species, and outbreaks of coral predators and coral disease, however in the past two decades climate change has emerged as the primary threat to coral reefs [2,6–8]. This was emphasised during the recent 2016–17 global marine heat wave, which led to the most extensive coral bleaching event in history, including remote and pristine reefs [9,10]. While dynamic systems like coral reefs have an innate capacity for natural recovery [11,12], the frequency, intensity and severity of mass coral bleaching and extreme weather events is increasing [13,14], diminishing the time and capacity for recovery between catastrophic events [15]. Further, larval supply, settlement and recruitment of coral larvae [16–18] and post-settlement survival are often compromised by chronic or repeated disturbance events [19–22]. A lack of natural recruitment and insufficient time for recovery between disturbance events conspire to make natural recovery unlikely, or impossible in many locations. Combating habitat loss on multiple levels is likely to be the fundamental issue for ecologists and managers in the Anthropocene, which has led to an increasing impetus and interest in interventions that may boost the resilience of reefs, or aid in the preservation and restoration of coral reef structure and function [23,24].

Until very recently, marine conservation has favoured passive habitat protection over restoration. However, recent research has shown that optimal conservation outcomes should include both habitat protection and restoration [25]. Restoration is common practice in terrestrial ecosystems, and is an established management tool for coastal habitats such as wetlands [26] and shellfish reefs [27–29], but has remained controversial for coral reefs, both in academia and amongst marine managers. Critics of coral restoration have argued that (1) coral restoration detracts focus from mitigating climate change and other threats to the marine environment [9,30], and (2) is pointless unless it can restore reefs at the ecosystem scale [31]. Proponents of coral restoration counter (1) that interventions can serve to protect coral biodiversity in the short-term, while mitigation of large-scale threats such as climate change and water quality take effect [24], (2) are necessary for the recovery of endangered and rare coral species such as *Acropora palmata* and *A*. *cervicornis* in the Caribbean where natural population maintenance has broken down [32–34], and (3) increase environmental stewardship and interest in protecting coral reefs by including local communities in restoration projects [35–39]. Global temperature is predicted to increase for several more decades even in a zero-carbon emission scenario [40,41]. Thus, if effective, local-scale restoration action could potentially bridge the temporal gap between large-scale action on climate change and the substantial lag effects predicted for indirect management actions. Given that disturbed reefs are likely to suffer a reduction in genetic diversity due to large-scale disturbance events during this period [42–44], preserving coral species and genetic diversity through active restoration could ‘buy time’ for recovery following amelioration or the removal of stressors.

Despite widespread reservations (in particular in the scientific community), active coral restoration has been increasingly used as a tool to restore coral reefs at local scales, especially by the tourism industry [32,45–48]. However, owing to poor communication and collaboration between coral restoration practitioners, coral reef managers and scientists, a large proportion of coral restoration work to date has been undertaken with little or no scientific input or detailed monitoring. Therefore, a substantial proportion of coral restoration projects and methods have not been documented in the scientific literature. A paucity of documentation, coordination and sharing of knowledge reduces our ability to learn from past successes and failures, and increases the risk of repeatedly testing similar methods and hypotheses. To counteract this, we aimed to synthesise the available knowledge in a comprehensive global review of coral restoration methods. We augmented the data collected from a scientific literature search with information from sources outside traditional academia, directly targeting restoration practitioners with an online survey and accessed online sources for specific details about restoration methods and new developments. Our objectives are to provide a systematic review of the current methods of coral restoration, highlight common problems and potential areas of concern and identify knowledge gaps. In addition to this review, we have produced an online interactive database to act as a resource for coral restoration practitioners, coral reef managers and scientists (access here), linked throughout the document. Combined, these three outputs (review, database and visualisation) establish a baseline of the current state of knowledge of global restoration approaches, to inform future research directions and improve restoration on coral reefs.

## Methods

We assembled case studies and descriptions of coral restoration methods from four sources: 1) the primary literature (i.e. published peer-reviewed scientific literature), 2) grey literature (e.g. scientific reports and technical summaries from experts in the field), 3) online descriptions (e.g. blogs and online videos describing projects), and 4) an online survey targeting restoration practitioners (doi:10.5061/dryad.p6r3816). We included only those case studies which actively conducted coral restoration (i.e. at least one stage of scleractinian coral life-history was involved). This excludes indirect coral restoration projects, such as disturbance mitigation (e.g. predator removal, disease control, etc.) and passive restoration interventions (e.g. enforcement of control against dynamite fishing or water quality improvement). It also excludes many artificial reefs, in particular if the aim was fisheries enhancement (i.e. fish aggregation devices), and if corals were not included in the method. To the best of our abilities, we avoided duplication of case studies across the four separate sources, so that each case in our review and database represents a separate project.

More than 40 separate categories of data were recorded from each case study and entered into a database. These included data on (1) the information source, (2) the case study particulars (e.g. location, duration, spatial scale, objectives, etc.), (3) specific details about the methods, (4) coral details (e.g. genus, species, morphology), (5) monitoring details, and (6) the outcomes and conclusions (S2 File). While our expanded search enabled us to avoid the bias from the more limited published literature, we acknowledge that using sources that have not undergone rigorous peer-review potentially introduces another bias. Many government reports undergo an informal peer-review; however, survey results and online descriptions may present a subjective account of restoration outcomes. To reduce subjective assessment of case studies, we opted not to interpret results or survey answers, instead only recording what was explicitly stated in each document (sensu [49,50].

### Primary literature

We used multiple search engines to achieve the most complete coverage of the scientific literature. First, we searched the scientific literature using Google Scholar with the keywords “coral* + restoration”. Because the field (and therefore search results) are dominated by transplantation studies, we then conducted separate searches for other common techniques using “coral* + restoration + [technique name]”. This search was further complemented by using the same keywords in ISI Web of Knowledge (search yield n = 738). We then manually selected studies that fulfilled our criteria for active coral restoration described above (final yield n = 221). In those cases where a single paper describes several different projects or methods, these were split into separate case studies. Finally, we consulted prior reviews of coral restoration to obtain case studies from their reference lists.

### Grey literature

While many reports appeared in the Google Scholar literature searches, we also consulted The Nature Conservancy (TNC) database of reports for North American coastal restoration projects (http://projects.tnc.org/coastal/). This was supplemented with reports listed in the reference lists of other papers, reports and reviews, and during our online searches (n = 30).

### Online records

Small-scale projects conducted without substantial input from researchers, academics, non-governmental organisations (NGO) or coral reef managers often do not result in formal written accounts of methods. To access this information, we conducted online searches of YouTube, Facebook and Google, using the search terms “Coral restoration”. We used information provided in videos, blog posts and websites to describe further projects (n = 48). Due to the unverified nature of such accounts, we have limited the data collected from these online-only records compared to peer reviewed literature and surveys. At the minimum, the location, the methods used and reported outcomes or lessons learned were included in this review.

### Online survey

In order to access information from projects not published elsewhere, we designed an online survey targeting restoration practitioners. The survey consisted of 25 questions querying restoration practitioners regarding projects they had undertaken (S1 File) under JCU human ethics H7218 (following the Australian National Statement on Ethical Conduct in Human Research, 2007). These data (n = 63) are included in all calculations within this review, but are not publicly available to preserve the anonymity of participants. Although we encouraged participants to fill out a separate survey for each case study, it is possible that participants included multiple separate projects in a single survey, which may reduce the real number of case studies reported.

### Data analysis

Due to the high heterogeneity of information available from such a diverse range of sources, we were precluded from performing quantitative statistical meta-analyses. Instead, this is a qualitative review using summary statistics to evaluate and contrast different restoration method outcomes. Percentages, counts and other quantifications from the database refer to the total number of case studies with data in that category. Case studies where data were lacking for the category in question, or lack appropriate detail (e.g. reporting ‘mixed’ for coral genera) are not included in calculations. Many categories allowed multiple answers (e.g. coral species); these were split into separate records for calculations (e.g. coral species *n*). For this reason, absolute numbers may exceed the number of case studies in the database. However, percentages reflect the proportion of case studies in each category. We used the six objectives outlined in [51] to classify the objective of each case study: (1) Accelerate reef recovery post-disturbance, (2) Reestablish a self-sustaining, functioning reef ecosystem (3) Mitigate anticipated coral loss prior to a known disturbance, (4) Reduce population declines and ecosystem degradation, (5) Provide alternative, sustainable livelihood opportunities, (6) Promote coral reef conservation stewardship, with an additional two categories: (7) scientific research, and (8) ecological engineering. We used Tableau to visualise and analyse the database (Desktop Professional Edition, version 10.5, Tableau Software). The data have been made available following the FAIR Guiding Principles for scientific data management and stewardship [52]. Data available from the Dryad Digital Repository downloaded here (https://doi.org/10.5061/dryad.p6r3816), and visually explored here.

### Defining restoration

The Society for Ecological Restoration International Science & Policy Working Group [53] defines **restoration** as “the process of assisting the recovery of an ecosystem that has been degraded, damaged, or destroyed”. Further, “restoration attempts to return an ecosystem to its historic trajectory”. Restoration projects ideally require no attendance once they are mature. Currently for coral reefs, the term restoration is used to encompass both ‘restoration’ and ‘rehabilitation’; with the latter emphasising “the reparation of ecosystem processes, productivity and services…” without meaning a return to pre-existing biotic conditions, and often requiring some attendance. A **restored ecosystem** “contains sufficient biotic and abiotic resources to continue its development without further assistance or subsidy”.

These definitions highlight one of the fundamental disconnects between the field of ecological restoration, developed largely in the terrestrial realm, and coral restoration. The International Principles and Standards for the Practice of Ecological Restoration [54] promote the use of a reference ecosystem as a model or target used to assess progress toward restoration of a local ecosystem. While recovery of endangered species (e.g. the *Acropora* sp.) does not fit this view of ecological restoration it has driven the development of one important technique used in coral reef restoration and is included in this review. The confounding of the aims of a project and monitoring to document successful endangered species recovery versus coral reef restoration is one source of confusion that complicates a review of the field.

Restoration can be passive or active, whereby **passive restoration** (also ‘natural regeneration’ or ‘indirect restoration’) “relies on increases in individuals, without direct planting or seeding, after the removal of causal factors alone”, while **active restoration** (also ‘direct restoration’, and often shortened to just ‘restoration’) relies on reintroductions or augmentations [54]. Broadly speaking, these two types of restoration also correspond to the level of degradation sustained by the environment, where passive restoration can be applied to sites with less damage, and active restoration is considered necessary in areas where unassisted natural recovery is unlikely. Finally, an **intervention** is the action **“**undertaken to achieve restoration, such as substratum amendment, exotics control, habitat conditioning, reintroductions” [54].

In this review, we have excluded passive restoration methods such as predator removal (e.g. crown-of-thorns starfish and *Drupella* control), unless they were conducted in conjunction with active restoration (e.g. macroalgal removal combined with transplantation). Instead, we review active restoration methods which reintroduce coral (e.g. coral fragment transplantation, or larval enhancement) or augment coral assemblages (e.g. substrate stabilisation, or algal removal), for the purposes of restoring the reef ecosystem. In the published literature and elsewhere, there are many terms that describe the same intervention. For clarity, we provide the terms we have used in the review, their definitions and alternative terms (Table 1).

**Table 1:** The terms for restoration methods used in the review, their definitions and other common terms. Categories are not mutually exclusive as some methods are often combined.

Method	Definition	Other common terms
ASEXUAL PROPAGATION METHODS		
Direct transplantation	*Transplanting coral colonies or fragments without intermediate nursery phase*	Coral tipping, post-disturbance repair
Coral gardening	*Transplanting coral fragments after an intermediate nursery phase*	Population enhancement, asexual propagation, *
Coral gardening—Nursery phase	*Transplanting coral fragments with an intermediate nursery phase (used to describe case studies that only detail the nursery phase)*. *Nurseries can be in situ (on the reef) or ex situ (flow through aquaria)*. *Note that following the above definition of restoration*, *a coral nursery does not constitute restoration*, *until outplanting has occurred*.	
Coral gardening—Transplantation phase	*Transplanting coral fragments with an intermediate nursery phase*, *including outplanting juveniles raised in the nursery (used to describe case studies that only detail the transplantation phase)*	Outplanting
Coral gardening—Micro-fragmentation	*Transplanting micro-fragments from corals*, *with an intermediate nursery phase*	Re-skinning
SEXUAL PROPAGATION METHODS		
Larval enhancement	*Using sexually derived coral larvae to release or outplant at restoration site*, *after intermediate holding phase which can be in- or ex-situ*	Larval propagation, sexual propagation, larval seeding, assisted breeding
SUBSTRATUM ENHANCEMENT METHODS		
Substratum addition—Artificial reef	*Adding artificial structures for purposes of coral reef restoration*	Engineered/artificial structures, various brand names (e.g. BioRock, EcoReef, ReefBall, Mars Spiders)
Substratum stabilisation	*Stabilising substratum to facilitate coral recruitment or recovery (often combined with artificial reefs and transplantation of coral fragments)*	
Substratum enhancement—electric	*Enhancing artificial substrata with an electrical field or direct current*	Electrochemically formed structures, mineral accretion, BioRock
Substratum enhancement—Algae removal	*Enhancing substrata by removing macroalgae*	

* In some geographic locations (primarily Caribbean, due to focus on endangered species recovery) coral restoration is synonymous to coral gardening (e.g. Fragments of Hope Coral Reef Replenishment Manual, Bowden-Kerby 2014)

## Coral restoration in a changing world

### Where, how and why is coral restoration occurring?

**Fig 1:**
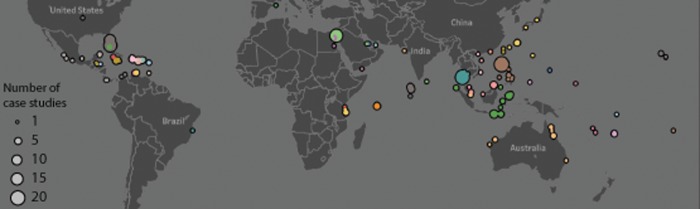
Location of coral restoration case studies included in the review. Restoration case studies occur in 56 countries, with most countries that have substantial coral reef area having at least one case study. Data points are coloured by country.

We identified 362 case studies on coral restoration, of which 221 were from the scientific literature, 78 were sourced from the grey literature (i.e. reports and online descriptions), and 63 were responses to our survey for restoration practitioners. Restoration projects occurred in 56 countries (Fig 1), with the majority of projects conducted in the USA (Florida, Hawaii), Philippines, Indonesia and Thailand (together representing 40% of projects). Ten categories of coral restoration are represented in the database, with the majority of these involving coral fragmentation or transplantation of coral fragments (68%).

Overall, the two primary objectives reported by practitioners were ‘scientific research’ (44%) and ‘accelerate recovery post-disturbance’ (38%, online visualisation). However, the objectives stated for each project differed markedly between data sources; where 65% of peer reviewed articles stated scientific research as their main objective, compared to 18 and 8% for the grey literature and surveys respectively. Instead, case studies from the grey literature and surveys mainly reported a desire to 'accelerate recovery post-disturbance’ (59 and 40% respectively). This echoes the findings from a recent review based on mostly published literature, where the motivations behind restoration were evaluated [55]. They found that most published (i.e. peer-reviewed) coral reef restoration research is focused on improving the restoration approach and answering questions of ecological concern (which corresponds to our category of ‘scientific research’). Similarly, Hein and colleagues [56] found that 60% of coral transplantation studies focused on evaluating the ‘biological response to transplantation’ (coded as scientific research in our study), while remaining 40% were primarily aimed at ‘accelerate recovery post-disturbance’ or ‘re-establish a self-sustaining, functioning reef ecosystem’.

### Temporal and spatial duration of restoration projects

**Fig 2:**
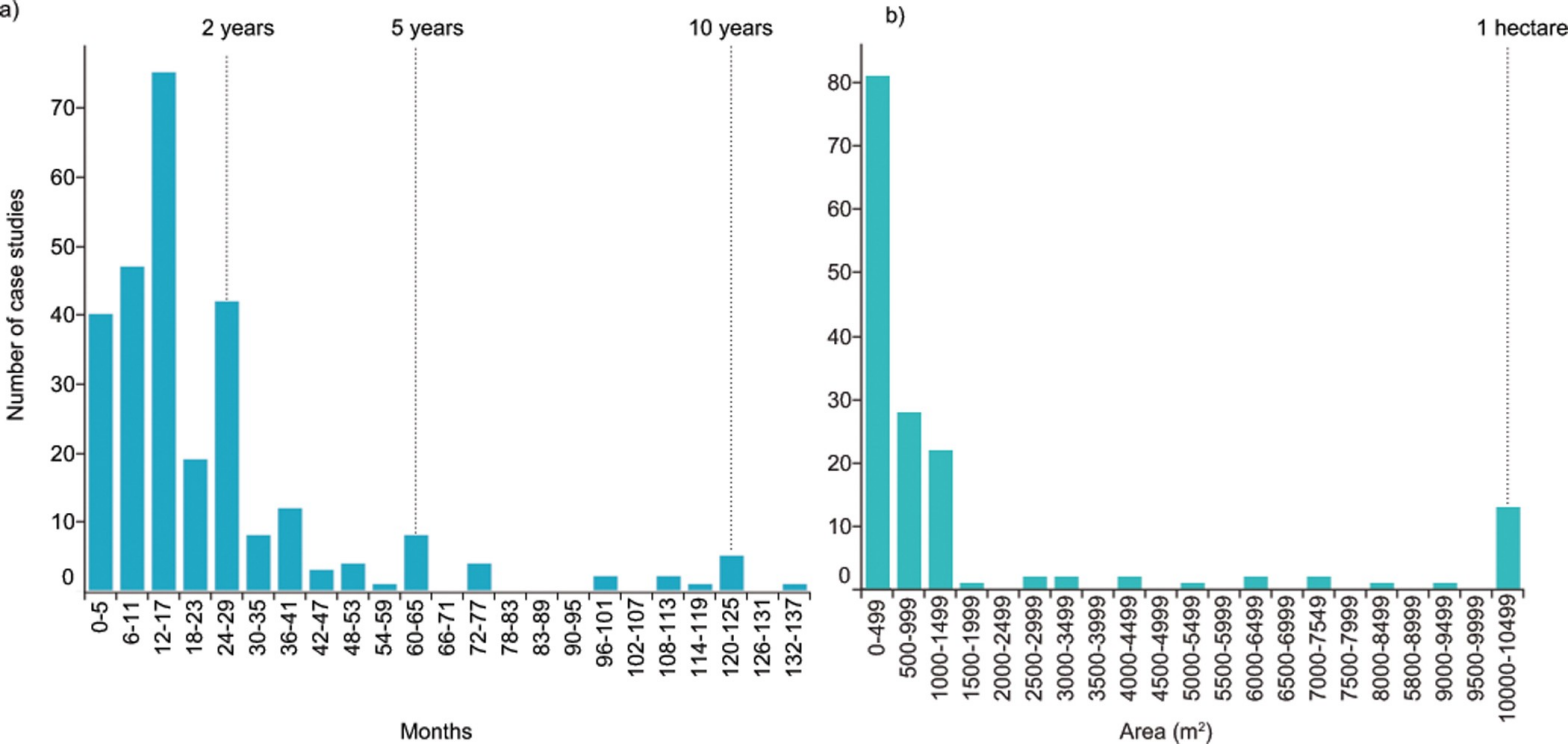
**The a) temporal and b) spatial scale of coral restoration projects included in the review.** Note that the x-axis in both panels have been truncated for visualisation purposes. Full figure can be viewed in the online visualisation.

Coral restoration case studies are dominated by short-term projects, with 60% of all projects reporting less than 18 months of monitoring. Overall, the median length of projects was 12 months, but this varied between project types. Survey respondents (i.e. coral restoration practitioners) tended to report longer projects (median 24 months), while grey literature and peer-reviewed projects both reported a median monitoring period of 12 months (Fig 2). The inconsistency between data sources may be explained by the relatively short time period available for most research projects (e.g. student projects and one year funding cycles), and the pressure to publish results quickly. In contrast, survey respondents may be more likely to report on the entire duration since restoration activities began (i.e. ‘time on the reef’). Similarly, most projects were spatially small, with a median size of restored areas of 100 m^2^ (Fig 2). Research projects published in the peer-reviewed literature reported a median spatial scale of 300 m^2^, while survey respondents reported larger spatial scales (median 500 m^2^). Median size of projects in the grey literature was 47 m^2^. Median values were used to describe spatial and temporal scales due to a substantial right-side (positive) skew in both data sets, with long tails.

Although monitoring most often occurred in a 12-month time frame, disturbances on reefs are stochastic. While most corals will experience a major bleaching event, destructive storm or disease outbreak in their lifetime, it is entirely feasible for at least 12-months to pass without these stressors. A mismatch between relatively short monitoring times and the temporal scale at which disturbances occur may artificially inflate the growth or survival rate. For example, Fadli *et al*., [57] described a successful restoration project in Indonesia, where coral cover, diversity and fish abundance improved dramatically on artificial reef modules after three years of deployment. However, almost 100% of these corals died in a bleaching event approximately six months after the conclusion of the study. While these authors reported this event in their publication, other practitioners may not know a major mortality event occurred if it happened after the project monitoring ceased, or have little incentive to publish a failed experiment. We argue that short monitoring times are problematic and may inflate the apparent survival rates of corals, as the likelihood of significant stress events causing mortality should increase over time. While there was no evidence of survival declining with increasing length of studies in the data, this could reflect the relatively low numbers of studies exceeding 12 months of monitoring. Further, mortality tends to be highest among early life stages and decreases as corals grow larger and older [58].

The longest monitoring period reported in our data set was 12 years for a transplantation project [59]. Studies that lasted 10 years or more (n = 5) tended to be described in reports based on monitoring programs for artificial reefs or restoration sites with transplanted corals; these also tended to be larger in spatial scale (>1,000 m^2^) than the short-term studies. Similarly, studies with a spatial scale greater than 1 hectare (10,000 m^2^, n = 17) were mainly monitoring projects of artificial reefs or coral transplantation sites. Unfortunately, despite being long-term projects of larger spatial scales, only two of these projects reported survival of corals (average survival 80%).

### Corals used in restoration projects

**Fig 3:**
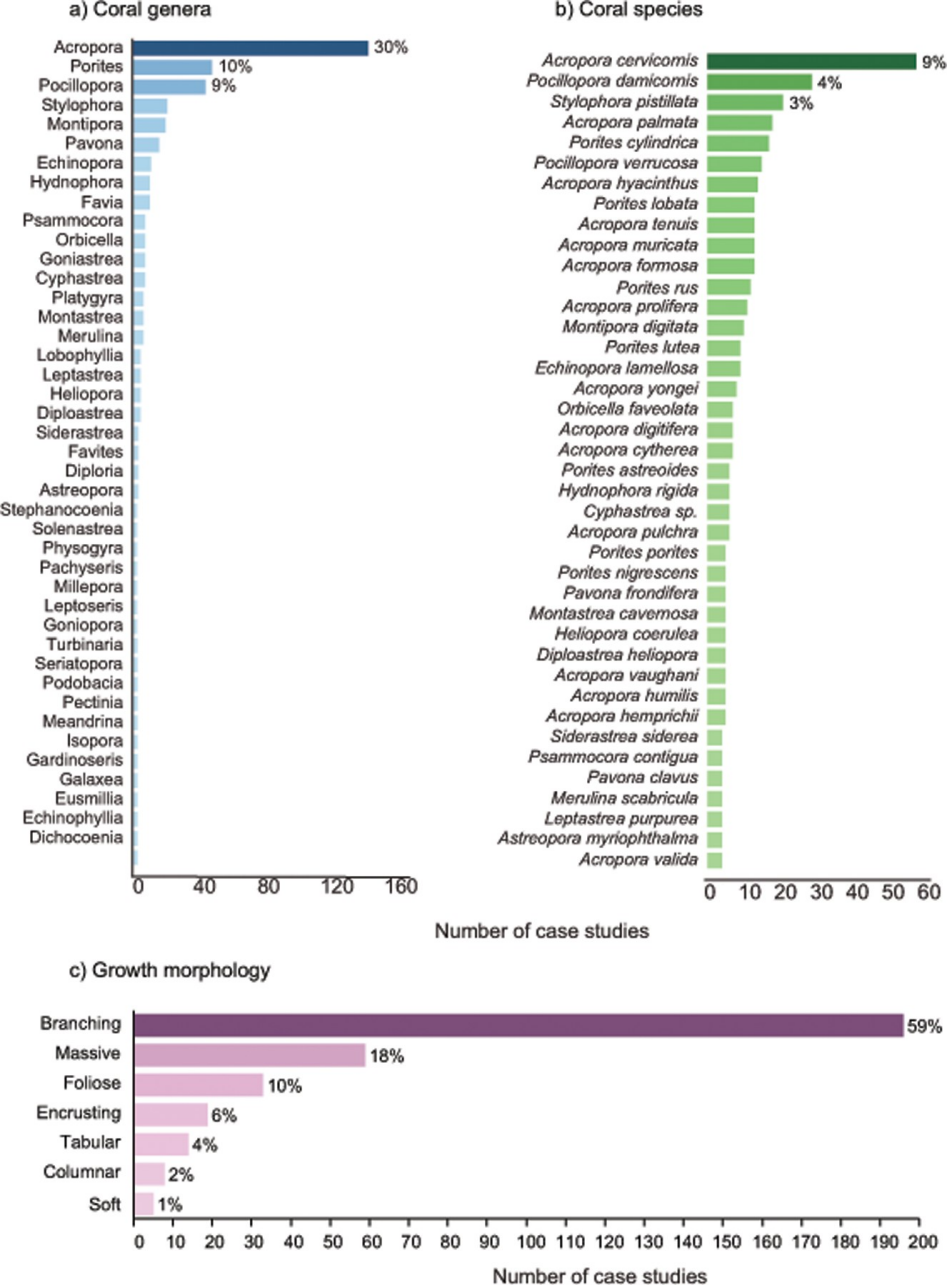
**The a) species b) genera and c) growth morphologies of corals used in coral restoration projects**. Note: The y-axis for genera and species is substantially truncated for visual purposes. The complete species list can be viewed in the online database. A large proportion of survey respondents did not report species or genera (but opted for ‘mixed’). The numbers reported here are therefore from the total number of case studies that reported on species or genera

Overall, coral restoration projects focused primarily on fast-growing branching corals (59% of studies). Almost three quarters (72%) of case studies reported using more than one coral species in their restoration projects, while the remaining 28% used a single species. A diverse range of species are thus represented in the dataset, including a total of 229 different species from 72 coral genera (Fig 3). A third of projects (30%) involved the coral genus *Acropora*, and 9% of studies included a single species–*Acropora cervicornis* [60–62]. Among all the published case studies, the top five species used in restoration projects were *Acropora cervicornis*, *Pocillopora damicornis*, *Stylophora pistillata*, *A*. *palmata* and *Porites cylindrica* (32% of studies, Fig 3). The focus on corals from the genera Acropora and Pocillopora is similar across all datasources. Much of the focus on *A*. *cervicornis* and *A*. *palmata* is likely to have resulted from these important reef-forming species being listed as threatened on the United States Endangered Species Act (71 FR 26852) and as Endangered on the International Union for Conservation of Nature Red List of Endangered Species (IUCN 2018).

The average survival rate of restored corals was 66% (± SEM 2.2%). When projects were subdivided by coral genera, survival tended to fall between 60–70% for the most commonly used genera (i.e. >10 case studies with survival data on that genus). While the average survival of some genera exceeded 90%, these estimates were based on no more than three case studies, suggesting that seemingly highly suitable genera are still poorly supported by available information, and further replicated studies (particularly of the well-performing genera) are needed. We refer the reader to supplemental information (S3 File) for more information on species and genera specific survival. The similarity in average survival between coral genera suggests that coral survival and success in restoration projects is less linked to individual coral species or genera used, and highlights the critical role of environmental conditions in shaping the outcome of restoration projects (see also [63]). To place these values in context within the broader restoration ecology field, an average survival of 66% is substantially higher than that reported in terrestrial ecological restoration, where outplant success tends to fall below 50% [64,65]. Further, a recent review of marine coastal habitat restoration in Australia highlighted that restoration in many marine ecosystems often report average survival of less than half of outplanted individuals. For example, 60% of seagrass restoration projects report <25% survival of seedlings [66].

## Restoration methods

We identified ten main coral restoration methods or techniques. We interrogated the database to evaluate the effectiveness of each method, and techniques associated with each intervention. These data are presented visually in our interactive database (access here), and described in detail in S3 File.

### Direct transplantation

The earliest and most common method of coral restoration involves the direct transplantation of coral fragments, from a donor to a recipient reef. There are 94 descriptions of direct transplantation in the review database, representing 20% of all records. This method is most common in programs aimed at salvaging corals from planned construction activities that would otherwise destroy or disturb the colonies [32,67–76]. Overall, direct transplantation studies reported an average survival of 64%, with 20% reporting >90% survival of transplanted corals (interactive database). Direct transplantation has primarily involved fast-growing corals, with more than three-quarters of case studies using branching coral morphologies. While there is an assumption that transplanted corals will attract other reef species, there is little evidence to support the notion of enhanced colonization by other reef species in the immediate vicinity of coral transplants [77]. Experiments designed to test the efficacy of transplanting corals are rare (as they are for many other methods), which hinders our ability to explore this further.

### Coral gardening

Continuous harvesting of coral fragments may have detrimental effects on donor corals and populations. In response to this, a more sustainable model has been developed where coral recruits or small fragments are raised in intermediate nurseries, prior to outplanting on restoration sites. The nursery phase protects corals from damaging conditions during their most vulnerable stages, with the intention of planting them onto damaged reefs once they have reached a size threshold at which their post-transplantation survival is higher [78]. In addition, once fragments have reached a suitable size they can be broken into smaller pieces, and these can be grown in the nursey, multiplying the number of fragments available to outplant. In this review, 48% of case studies involved coral gardening, with a majority of records focusing on the transplantation phase of the concept (transplantation phase 24%, nursery phase 16%, both phases 8%). Corals were raised in either field-based (*in situ*), or land-based *(ex situ*) nurseries, depending on local conditions. Practitioners advocating the use of a coral nursery phase for reef restoration highlight improved growth and survivorship rates of fragments, compared to direct transplantation. While some projects do report high survival (e.g. >75%; [79]) this is not echoed in our dataset, where coral gardening studies exhibited an average 66% survival in the outplanting phase, compared to 64% survival in direct transplantation studies which lacked an intermediate nursery phase.

### Micro-fragmentation

Less than 5% of transplantation studies have been conducted with corals characterised by slow growing life histories. Massive corals have largely been overlooked, mainly due to their slow growth and thicker skeletons, which are less amenable to fragmenting [80]. However, researchers from Mote Marine Laboratory have developed a ‘micro-fragmentation’ technique that enables massive and encrusting corals to be produced and outplanted using concepts developed for coral gardening [80,81]. A diamond blade saw is used to cut small fragments (1 cm^2^) of massive corals, which are then mounted on tiles. After approximately 12 months, the fragments can either be further sub-divided to generate new micro-fragments or outplanted. Micro-fragments that are secured to reef substrates or dead coral skeletons in an array will readily fuse together to form a larger colony (termed ‘re-skinning’). The research outcomes show high survival and rapid growth of fragments [80,81].

### Genetic diversity in asexual propagation

If the goals of restoration are to include resilience to existing or future stresses, the consideration of genetic diversity is crucial [82]. Acroporids, which are used preferentially in asexual propagation methods, naturally reproduce asexually through fragmentation, so the recommended genetic diversity ratio reflects the proportion of unique genotypes per number of colonies sampled in a specific stand or thicket [83]. The clonal processes preferentially used in coral gardening inherently limits the proliferation of different genotypes and hence resilience. Assisted fertilisation [84] or creating nursery stocks from the larvae of brooding corals [85] could be valuable tools for maintaining genetic diversity in coral gardening. The NOAA recovery plan [86] suggests a target genetic diversity ratio of 0.5 for both *A*. *cervicornis* and *A*. *palmata* [83].

### Larval enhancement

Larval enhancement methods aim to increase rates of coral fertilisation, larval survival and recruitment. Fertilisation may be limited on reefs with low coral cover or asynchronous spawning. Planktonic development of embryos and larvae may result in a high proportion of coral larvae being swept away from reefs and therefore failing to settle or recruit [16,17,87]. Harnessing the power of corals to produce millions of young by reducing these early life history mortalities may be the most likely way to scale up restoration beyond present small-scale solutions. Six studies (1.3%) describe the relatively new method of larval enhancement, grouped into two main types: one where larvae are settled on artificial structures, and one where larvae are settled directly onto the reef.

The first method uses harvested gametes with embryos reared ex-situ, which are subsequently settled onto a range of artificial structures developed to improve post-settlement survival rates [88,89]. For example, concrete tetrapods were recently ‘seeded’ with Caribbean *Favia fragum* larvae that had been fertilised and reared *ex situ* [90], with multiple recruits per unit. The ‘seeding units’ were scattered onto a degraded reef area, after a four-week juvenile coral rearing period. Approximately 10% of settled larvae survived, and 56% of seeding units harboured at least one *F*. *fragum* individual one year after deployment. The authors concluded that the main advantage of this method over others is the speed of outplanting compared to methods which attach coral fragments individually. The second larval enhancement technique also uses coral gametes are collected during spawning but embryos and larvae are subsequently reared in holding tanks or on the reef, after which larvae are released directly onto the reef in enclosures that retain them over the target substrate during the settlement period [58,91,92]. Recently, longer-term replicated larval enhancement and recruitment trials have been completed successfully on highly degraded reef areas in Northern Luzon, Philippines [58,93]. This work demonstrated that mass larval settlement on degraded reef areas (4 x 6 m) can significantly enhance recruitment and re-establish a breeding population of *A*. *tenuis* colonies after three years.

### Artificial reefs

About one fifth of the projects we reviewed (21%) we reviewed involved the creation or addition of substratum, such as artificial reefs. The creation of substratum involves structures that are placed on the seabed deliberately, sometimes to mimic characteristics of a natural reef, or for the purpose of increasing potential habitat for reef fauna, fisheries production, recreational diving opportunities, or the prevention of trawling. In many cases, artificial reefs are deployed in conjunction with other methods, such as coral transplantation [57, 94,95]. In the past decade, Mars Incorporated has developed a modular approach to restoring corals that is particularly suitable for deployment on unstable substrate. The technique uses small, modular, open structures (termed ‘spiders’) made from steel bars fabricated into a hexagonal structure resembling a spider, which is then coated in a rough textured protective coating (consisting of resin and coarse sand) for coral to adhere to. The technique builds upon similar artificial reef techniques and frames developed in the Indian Ocean and has been used in Indonesia to remediate reefs affected by dynamite and cyanide fishing [96]. Note that a large proportion of artificial reefs created for fisheries augmentation (i.e. fish aggregation devices) were not included here as they do not directly involve corals (see S3 File). However, artificial reef projects that transplanted corals, and monitored survival, reported an average of 66% survival.

### Substratum stabilisation

The direct physical restoration of damaged substratum mostly involves stabilising rubble in an area that has been affected by storms or ship groundings. The rationale for stabilisation is that survival rates for coral recruits are low on loose substratum [97]. While substratum stabilisation has been used relatively often in US territorial waters, funded by insurance claims following ship-strikes, there is a paucity of published literature that clearly describes methods and techniques (4% of case studies in this review). The most common method is to install mesh or netting over the rubble to prevent further movement. This is generally a precursor to the transplantation of corals onto the damaged area [97] and/or additional deployment of artificial structures. Substrate stabilisation projects which reported survival of corals (n = 5) described an average survival of 80%.

### Substratum enhancement with electricity

The aim of the technique is to mimic the chemical and physical properties of reef limestone, by encouraging the precipitation of calcium and magnesium on artificial substrata [98]. A direct electrical current is established between electrodes, and calcium carbonate and magnesium hydroxide precipitates at the cathode, while oxygen and chlorine are produced at the anode [99]. The purpose of this mineral accretion is to potentially increase the calcification of coral polyps, thereby boosting colony growth and resilience to stressors. The technique has been controversial and experiments attempting to verify its effectiveness have had varied outcomes. Sabater and Yap [100] described increased growth and attachment in *P*. *cylindrica* fragments when connected to a setup similar to that described by Goreau and Hilbertz [101]. A range of other studies have described increased survival of fragments on mineral accretion frames [102–106]. However, multiple experiments have failed to describe similar positive effects of exposing coral fragments to an electrical field. For example, Romatzki [107] found that *A*. *pulchra* and *A*. *yongei* coral fragments exposed to similar strength electrical currents as those described by previous researchers grew slower than control colonies. Similarly, Borell [108] described negative effects on growth of one species of coral (*A*. *yongei*) but positive effects on another (*A*. *pulchra*) growing on a cathode, suggesting that results may vary even between congeneric coral species. The disagreement between studies prohibits clear conclusions about the mineral accretions method.

## Conclusions and recommendations

This review has summarised four decades of coral restoration projects and research, and to our knowledge is the most comprehensive review of existing coral restoration techniques available so far. The accompanying interactive database (accessed here) provides an evidence-based resource for researchers, managers and practitioners to draw from and build on. We document successes and failures of coral restoration in the past decades as reported by scientists, managers and practitioners. Coral restoration has shared some of the common ‘growing pains’ associated with ecological restoration more broadly. For example, Lake [109] highlighted the five obstacles to scaling ecological restoration of freshwater lakes and rivers in Australia, and there is an almost complete overlap with issues emerging from coral restoration projects reviewed during this study. (1) The reluctance of resource managers to undertake large and long term restoration projects, which is evident in our data from the small size and short timeframes of a majority of projects. (2) Poor design of many restoration projects (e.g. many projects lack experimental controls and adequate replication, and have poorly chosen reference systems etc.). (3) A lack of adequate and ongoing monitoring of projects; combined with (4) a lack of reporting on the progress and outcomes of projects. Finally, (5) many projects have challenges associated with increasing spatial and temporal scale.

The science and practice of ecological restoration (including coral reef restoration) has much to gain from the science of ecology; and there is some evidence that this is happening. Conversely, in our rapidly changing planet, ecologists are learning from restoration projects. Similarly, a broader awareness of lessons learned by restoration practitioners in the terrestrial and freshwater realms as well as in other marine habitats will speed the transfer of knowledge. This will speed the transition or coral restoration from small to ecological scales, a transition that is critical to the successful application of coral restoration for reefwide resilience. However, we highlight that it is critical to not view restoration as a replacement for meaningful action on climate change. While there is some evidence to suggest that local management actions can boost the resilience of corals to more substantial threats, including climate change [110], small scale solutions are unlikely to match the scale of the climate change crisis [31,111].

Most methods described in this review have documented successful coral growth, and relatively high levels of survival. We direct the reader to each specific section for descriptions of the potential application and limitations associated with each method. We also highlight two challenges, which if resolved, present opportunities for improved future project success. These include 1) a lack of clear and achievable objectives, 2) a lack of appropriate and standardised monitoring and reporting and, 3) poorly designed projects in relation to stated objectives.

### Objectives and monitoring

Objectives for coral restoration usually have a broad ecological scope that aligns with principles of reef resilience [51]. For example, the stated aims of coral restoration usually focus on accelerating reef recovery [59,112,113], re-establishing a functioning reef ecosystem [46,114], or mitigating population declines and endangered species management [115]. However, in some projects there is a clear mismatch between stated objectives and what is actually measured during monitoring of outcomes. For example, of projects with an ecological objective in this review (n = 129), 45% did not measure any relevant metrics to evaluate project success relating to that objective (e.g. stated objective is to restore ecological function of a reef, yet monitoring is exclusively focused on biological metrics of individual coral fragments). This mismatch not only prevents scientific evaluation of project outcomes, but also carries the risk of reducing public and academic support for coral restoration in general, by building expectation and then failing to provide evidence to evaluate success or failure.

Coral restoration projects targeted at reef recovery, should have the re-establishment of breeding populations as a fundamental aim [54,58,116]. Such populations should not need ongoing interventions, and should enhance natural larval production and recruitment processes. The explicit objective of self-sustaining breeding populations is a key missing component in almost all coral reef restoration projects, and evidence supporting that the objective is met is rarely monitored. This is implicit in the median monitoring period of 12 months which is far less than the length of time required for corals to reach reproductive maturity. In this review, for example, we found that outcomes of coral restoration projects are largely monitored with biological metrics of corals (e.g. growth and survival). Coral growth and survival were cited as outcomes in approximately 60% of the published literature, and constituted the majority of outcome metrics in the responses to survey questions. This echoes what has been found in previous reviews [51,55]. Other popular biological metrics used as outcomes included the condition and self-attachment of fragments. While these metrics are important for assessing the feasibility of coral restoration and for refining techniques, ecological monitoring is required to assess the ecological outcomes of restoration efforts and whether or not the initial aims and objectives are met. Appropriate metrics would also enable better assessments of the ecological and demographic processes occurring at restored areas [117,118], and thus inform adaptive management of restoration efforts in the longer term.

By necessity, we have used survival of corals to compare different studies in this review. While useful to assess the effectiveness of short-term performance of each method, it is becoming increasingly clear that this is not always a relevant metric to assess the long-term success of restoration projects. First, published survival data are potentially biased towards studies reporting higher survival. There is limited incentive to publish or report failed restoration projects for researchers, practitioners and managers alike, and this may be influenced by concerns about discouraging funders and the general public. Second, shorter monitoring times may artificially inflate survival data further by not reflecting the true long-term fate of restored corals. Finally, for the growing number of projects using sexual propagation as a source of coral propagules, which naturally have low survivorship [58], it becomes an almost meaningless metric. This further highlights the need for a more holistic suite of reef-wide ecological monitoring tools aligned to project objectives rather than relying solely on simple coral biological metrics.

Socio-cultural and economic outcomes should also be assessed as part of coral restoration objectives, such as whether or not coral restoration can promote alternative livelihoods [32,119], or promote local conservation stewardship [120,121]. As in other forms of management, adequate stakeholder involvement in planning and implementation of restoration efforts is likely to significantly influence project outcomes [39,96,113,122,123]. Finally, practitioners should involve local communities as much as possible throughout the planning and implementation of the project to ensure local ownership, i.e. identification with and stewardship of restoration projects, to ensure that the restoration efforts are aligned with local objectives and the restored coral reef can flourish after active interventions have ceased.

While the need to broaden *what* is monitored is clear, there is also a need to standardise *how* outcomes of restoration projects are monitored. A lack of standardisation in how to record mortality, survival, and growth makes these metrics difficult to compare between studies. Even the basic unit of the organism in question lacks standardisation. Coral gardening projects often refer to “fragments” without quantifying the size (average, min, or max) of these units [32]. The same lack of precision applies to the term “coral colony”. This absence of specificity in defining fragments or colonies in terms of their size hampers our ability to quantify and compare survivorship. Survivorship of a small (e.g. 10 cm^2^) fragment or colony is expected to be far less than that of a 40 cm^2^ unit (e.g. [124,125] but see [61]), and yet, in most reports, percent survival of outplants is not clearly reported in accordance to size. The metric that best illustrates the lack of standardised reporting is growth of corals, where a multitude of creative metrics have been used–including linear extension (the summed length of each individual branch within a colony [62,115,126,127], height [128], ‘ecological volume’ [129,130], branch width [60,131], number of branches [132–134], basal width [135] as well as combinations of width, length, height and partial mortality, maximum colony diameter, number of branches, and virtually every other dimension one could think of. The metrics used have such little overlap that comparisons of overall growth among studies are difficult or impossible. Similarly, mortality or survival are often reported without explaining what specifically is being measured. Most studies use a simple binary scoring system (i.e. ‘live’, ‘dead’), while others quantify partial mortality of individual coral fragments (e.g. >0% live tissue remaining = live [61,62,97,136,137]). Established methodological approaches to coral demography [138] should be used which will not only help standardise metrics but also facilitate modelling approaches to assess the utility of the wide range of possible restoration approaches that exist. Appropriate metrics can then be used to aid management decision-making via calculating return on effort to further optimise practice [63].

While the lack of standardisation is problematic, it is an understandable consequence of numerous groups and practitioners operating in isolation, with little communication and with widely different objectives. This issue is now recognised, and groups like The Nature Conservancy’s Reef Resilience Network and the Coral Restoration Consortium are developing and sharing best practice guidelines for coral restoration worldwide. We further encourage researchers to publish ‘failed’ experiments and projects to benefit the rapidly developing field of coral restoration (the journal Restoration Ecology currently contains a single reference for coral restoration in their Setbacks and Surprises section; [139]). We suggest practitioners adopt the following guidelines for reporting the outcomes of restoration projects: 1) be explicit when describing how your metrics were calculated, and what the calculations are based on, 2) avoid inventing new metrics for simple demographic parameters, unless the new metric is a substantial improvement on existing methods. Refer to the published literature and use methods established in the broader field of coral ecology, rather than inventing new metrics that will isolate data within the relatively small realm of coral restoration literature, 3) avoid complex equations (i.e. multiple steps away from raw data) in favour of simple calculations (i.e. single or few steps away from raw data), and 4) communicate your observations and discoveries with the international scientific and practitioner community to make sure that no knowledge is lost.

The ideal monitoring program would be comprehensive and holistic, including ecological, social and economic metrics. However, all projects are limited in terms of funding and logistical capacity, and will most likely be unable to monitor the complete range of metrics. Ultimately, practitioners should adopt a monitoring program that is clearly linked to the stated objectives of the project. Often it may be appropriate to monitor projects in multiple phases; for example, short-term monitoring of biological metrics to evaluate method efficacy combined with long-term monitoring of ecological outcomes. Further, the use of proxies and indicator metrics may help reduce costs and time required. For example, coral cover and complexity may provide a suitable indicator of restored habitat value for other reef species. In addition, the number or proportion of breeding corals may be a more useful measure of restoration success than survival and growth of outplants. Proxies and indicators need to be thoroughly tested and evaluated before widespread use. Finally, local permit restrictions and conditions may ultimately shape monitoring programs more than idealised guidelines with a pure scientific perspective. For example, restoration projects in the Florida Keys National Marine Sanctuary are obliged to conduct extensive ‘fate-tracking’ of individual outplanted fragments through time, such that there is limited scope to conduct more extensive ecological monitoring. Further, the low-diversity and abundance of extant live corals on Floridian and Caribbean reefs compared to their Indo-Pacific counterparts, and the strong focus on the recovery of two endangered species (*A*. *palmata*, *A cervicornis*) may make some metrics less suitable. Clarity is needed on the distinction between metrics that address endangered species recovery and the place of those endangered species in the overall reef restoration. Practitioners should seek out local best practice guidelines wherever possible.

Many projects have stated ecological objectives, when in reality they are primarily aimed at social (e.g. local capacity building, stewardship) and or economic (e.g job creation, ‘edutourism’) objectives. Socio-economic objectives can be appropriate and important, and we argue against the notion that ecological objectives are the only relevant goals of restoration. However, much of the public and scientific distrust of coral restoration could stem from a mismatch between what is publicly portrayed as the objectives and goals of projects, and the executed reality. We encourage practitioners to state clear realistic objectives that are specific, measurable, achievable, realistic, and time-bound (SMART) [140] and to design projects and monitoring programs that are well aligned to those objectives, to avoid artificially inflating expectations.

### Future considerations

The future of coral reef restoration is likely to diverge towards two different scales; 1) small-scale site stewardship projects and 2) large-scale reef wide interventions. The former includes local projects with socio-economic objectives, such as those pioneered and led by tourism-industry and citizen scientists. While scale can be achieved through broad uptake by key stakeholders, these projects are likely to harness existing technologies to increase coral cover at select high value sites. The latter category is critical on an ecosystem scale and requires substantial spatial scaling up of projects, if restoration is to meet future challenges to coral reefs. Coral restoration is a rapidly changing field, and large-scale projects may need to be radically different from techniques described in this review. While many past projects have been relatively small, isolated and disconnected, reef-scale interventions will need to be multidisciplinary and are likely to require some degree of automation, and be highly coordinated and connected to match the scale of the problem. Of the techniques described in this review, few have demonstrated potential to be scaled up beyond a hectare of restored coral reef. The most scalable methods (i.e. beyond 1ha in a single project) appear to be techniques that use sexually derived propagules as a source for restored coral populations and communities.

### Environmental considerations

During compilation of this review, it has become evident that while coral restoration projects aim to solve the problem of habitat loss on coral reefs, some techniques may inadvertently contribute to the very problem they are trying to mitigate. For example, a majority of artificial reefs and structures are made from concrete, and 10% of studies which attach corals to the substrate used cement and concrete. The production of cement is responsible for 5–7% of global carbon emissions, mainly due to CO_2_ emissions during the calcination process of limestone, from combustion of fuels in the kiln, as well as from power generation [141]. The proportional contribution of coral restoration to the overall carbon footprint of concrete is of course negligible, however there is considerable irony in using a technique for restoration that directly contributes to climate change. Further, a substantial number of projects (~60%) use plastics to attach coral fragments to the substrate, primarily in the form of epoxy putty or cable ties. There are marine grade versions of both of these materials, although they are also likely to break down in the shallow, warm and high-UV environments of corals reefs. Other manufactured materials such as steel are also commonly used in coral restoration [76,78]. All of these materials can accumulate on reefs, potentially with unforeseen longer term consequences. However, while the growing problem of microplastics in the marine environment has been demonstrated to be detrimental to corals [142,143] it pales in comparison to the primary threat of climate change. As the field of coral restoration grows and spreads to more coral reefs around the world, we urge practitioners to use biodegradable alternatives, source local materials, employ local people and to avoid contributing to the problems facing coral reefs in the Anthropocene.

This review presents the most comprehensive summary of active coral restoration methods used to date, and combined with the online database, provides a resource for scientists, practitioners and managers. We have described coral restoration projects throughout the tropics, with a surprising diversity of coral species and morphologies used. While few projects have reported on ecological success, there is substantial evidence of our abilities to grow corals at smaller scales. Overall, the main techniques report similar average survival and growth of corals, so decisions on what techniques to use should be based on local conditions, cost, availability of materials and appropriateness based on stated objectives. There are ongoing refinements of techniques, with a growing focus on scaling up both spatially and temporally. Coral restoration methods and projects could be a component of resilience based management [66,144], along with water quality and fisheries management. However, one of the biggest drivers of coral reef decline is climate change. While many projects address this by propagating presumed heat-tolerant corals (i.e. those that survived recent bleaching events), coral restoration is ultimately not a replacement for meaningful management of reef resources and action on climate change.

## Supporting information

S1 File Reef restoration practitioners survey. (PDF)

S2 File Data collected from each case study. (DOCX)

S3 File Detailed information about coral restoration methods, technique and success. (DOCX)

S4 File PRISMA 2009 checklist. (DOCX)

S1 Fig PRISMA 2009 flow diagram. (DOC)
