# Supplementary material for: Coral restoration – A systematic review of current methods, successes, failures and future directions
Source: PLoS One. 2020 Jan 30;15(1):e0226631. doi: 10.1371/journal.pone.0226631 (PMC6992220; doi:10.1371/journal.pone.0226631)
Supplement: S1 File — (PDF) [file pone.0226631.s001.pdf]

Reef Restoration Practitioners Survey

Researchers at TropWATER, James Cook University, and Reef Ecologic, Australia, are collecting information about various techniques and methods used to restore coral reefs around the world. We plan to use this information to produce a review of current knowledge about coral restoration methods, so that we can learn from previous successes and failures, and better inform potential future restoration efforts.

Thank you for your time in filling out this survey, your knowledge will form an important part of this review.

Please note, you can remain anonymous throughout the survey, UNLESS you upload images as Google will automatically add your name to any uploaded file names.

*Required

TropWATER
Centre for Tropical Water and Aquatic Ecosystem Research

Reef Ecologic
For a better planet

To find out more about us, visit tropwater.org and reefecologic.org

Tropical Water Quality Hub
National Environmental Science Programme

This project is supported through funding from the Australian Government's National Environmental Science Programme.

1. **Consent Statement** *
I understand the aim of this research study is to review methods of active coral restoration. I consent to participate in this project, the details of which have been explained to me, and I have been provided with a written information sheet to keep (via email). I understand that my participation will involve an online survey and I agree that the researcher may use the results as described in the information email. If you haven't received the information sheet, it can be accessed here: https://goo.gl/bjaqiA
Mark only one oval.

- ( ) Yes, I consent to complete this online survey.
- ( ) No, I do not consent to complete this online survey. *After the last question in this section, stop filling in this form.*

2. **Where did your project take place?** *
Please name country and location (island/town)

____

3. **What is the name of the organisation?**
This question is optional. If you choose to leave this blank we will respect your anonymity.

____

4. **What type of organisation conducted the restoration project?** *
Choose all that apply
Tick all that apply.

- [ ] Government
- [ ] Business/Tourism operator
- [ ] NGO
- [ ] Research Institute
- [ ] Private
- [ ] Community group/Citizen Science
- [ ] Other: ____________________________

5. **Which groups of people or organisations were directly involved in planning or doing the restoration project?** *
Choose all that apply
Tick all that apply.

- [ ] Tourism, fishing or other businesses
- [ ] Local government agencies
- [ ] Marine park managers
- [ ] National government
- [ ] NGOs or community organisations
- [ ] Community organisations / citizen volunteers
- [ ] The lead organisation conducted all aspects of the restoration project
- [ ] Other: ____________________________

6. **In what practical ways did the restoration project address outcomes for people's well-being or livelihoods?** *
This includes both POSITIVE and NEGATIVE outcomes. Choose all that apply
Tick all that apply.

- [ ] Groups of people most likely to benefit or be affected by the project were directly involved in the design
- [ ] A risk assessment was undertaken that included social or economic issues (e.g. well-being, livelihood or legal impacts)
- [ ] The restoration project team included social researchers
- [ ] The project team applied for human and social research ethics approval
- [ ] The project did not directly address social or economic issues related to the outcomes of the restoration project.
- [ ] Other: ____________________

7. **What type of environmental data was collected before the intervention?** *
Tick all that apply.

- [ ] Specific surveys and reef monitoring was conducted
- [ ] Visual assessment (also photography/video)
- [ ] No prior assessment
- [ ] Other: ____________________

8. Please describe the methods used in your environmental monitoring (if any occurred) here:

____
____
____
____
____

9. **Why did you choose the specific site to conduct reef restoration?** *
Rank in order of importance: 1=most important, 5=least important
Mark only one oval per row.

	Not relevant	1	2	3	4	5
Site impacted by disturbance	◯	◯	◯	◯	◯	◯
Logistic reasons (i.e: close to dive resort, ease of access etc)	◯	◯	◯	◯	◯	◯
Legal reasons (i.e: could only get permits for this site)	◯	◯	◯	◯	◯	◯
Political reasons	◯	◯	◯	◯	◯	◯
Ecological reason (i.e: site important for connectivity/biodiversity etc.)	◯	◯	◯	◯	◯	◯
Other	◯	◯	◯	◯	◯	◯

10. If you selected other above, please describe here:

____

11. **What types of coral reef restoration methods have you used?** *

Choose all that apply.
Tick all that apply.

- [ ] Coral transplantation
- [ ] Substrate creation (E.g artificial reefs)
- [ ] Algae removal
- [ ] Substrate stabilisation
- [ ] Other: ______________________

12. **What was the objective of the restoration project?** *

Rank relevant objectives in order of importance: 1=most important, 5=least important.
Mark only one oval per row.

	Not relevant	1	2	3	4	5
Speed up reef recovery following a disturbance (e.g: replicating what existed before)	◯	◯	◯	◯	◯	◯
Restore the function of a degraded reef (e.g: without trying to replicate what previously existed)	◯	◯	◯	◯	◯	◯
Relocate corals prior to planned disturbance (e.g: jetty construction or development)	◯	◯	◯	◯	◯	◯
Reduce pressure on existing reefs	◯	◯	◯	◯	◯	◯
Creation of tourist attraction	◯	◯	◯	◯	◯	◯
Preserve particular species (e.g: a type of coral)	◯	◯	◯	◯	◯	◯
Provide alternative, sustainable livelihood opportunities	◯	◯	◯	◯	◯	◯
Provide opportunities for community/tourism involvement in coral reef conservation	◯	◯	◯	◯	◯	◯
Creation of new habitat	◯	◯	◯	◯	◯	◯
Scientific research	◯	◯	◯	◯	◯	◯
Other	◯	◯	◯	◯	◯	◯

13. If you selected other above, please describe here:

____

14. **Was restoration undertaken to address a specific disturbance? If yes, what was the disturbance?** *

(e.g: cyclone, tourism, ship grounding etc)

____

15. **Over what area of reef did you attempt restoration?** *

Mark only one oval.

- ◯ 100 m2 (example: 10 x 10 m, or half the size of a tennis court)
- ◯ 500m2 (example: 22 x 22m, or 2 times the size of a tennis court)
- ◯ 1,000m2 (example: 32 x 32m or 4 times the size of a tennis court)
- ◯ 10,000m2 (1 hectare, example: 100 x 100m, or 40 times the size of a tennis court)

16. **Can you explain what methods you used to attempt reef restoration?** *

Be specific where possible - Including tools and materials (glue/adhesives) used, substrate type (artificial/natural) etc.

17. **If your restoration methods involved corals, which types were used?**

(Choose all that apply)

Tick all that apply.

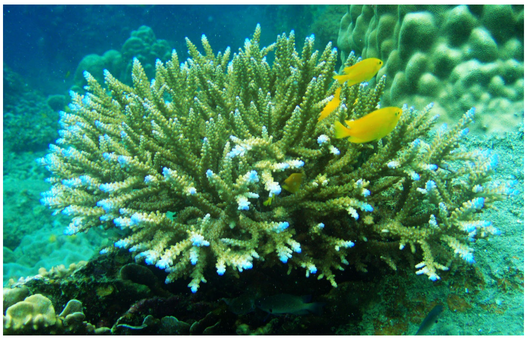

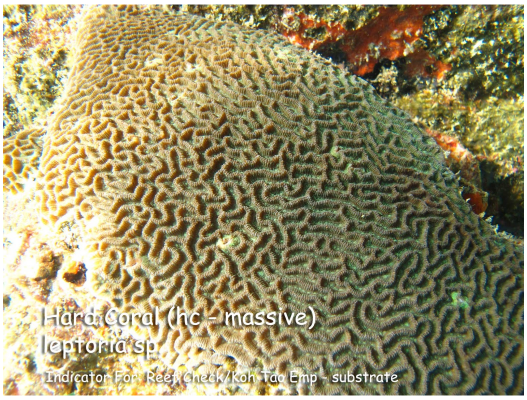

- [ ] Branching
- [ ] Massive

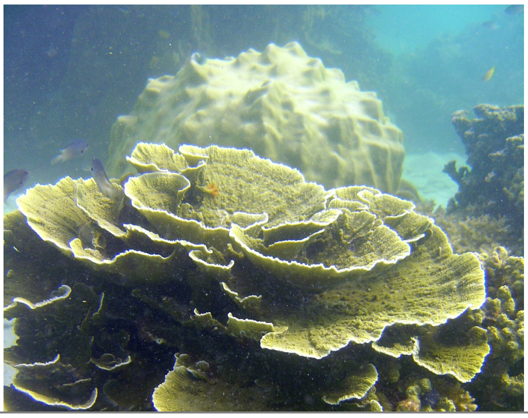

- [ ] Foliose
- [ ] Other:

18. **If your method involved transplantation of corals, what was the source of the coral transplants?** *

Choose all that apply
Tick all that apply.

- [ ] Already loose fragments (corals of opportunity)
- [ ] Whole colonies transplanted from nearby locations (i.e: same reef)
- [ ] Fragments taken from nearby colonies (i.e: same reef)
- [ ] Whole colonies transplanted from other locations
- [ ] Fragments transplanted from other locations
- [ ] Self sustaining nurseries
- [ ] Sexually produced coral larvae
- [ ] Did not transplant corals
- [ ] Other: ____________________

19. **After the initial reef restoration, did you undertake any further work (i.e: maintenance or monitoring)?** *

Please describe briefly what was done.

____

20. If you undertook monitoring and maintenance, how long did this occur (weeks/months/years)?

____

21. **How was the project funded?** *

Rank in order of financial contribution: 1=most important, 7=least important
Mark only one oval per row.

	Not relevant	1	2	3	4	5
Government	◯	◯	◯	◯	◯	◯
University/Research Institution	◯	◯	◯	◯	◯	◯
NGO/Charity organisation	◯	◯	◯	◯	◯	◯
Business	◯	◯	◯	◯	◯	◯
Private	◯	◯	◯	◯	◯	◯
Tourism	◯	◯	◯	◯	◯	◯
Other	◯	◯	◯	◯	◯	◯

22. **How long was the restoration project?** *

The time that active participation in the restoration project occurred. If ongoing, state how long the project has been going for.

____

23. **In your opinion, was this project successful?** *

Relative to your project objectives.
Mark only one oval.

- ◯ Yes
- ◯ Partial
- ◯ No

24. **What have been important lessons gained from your reef restoration project?** *

25. **Can we contact you for further information?**
Mark only one oval.

- Yes *After the last question in this section, skip to question 27.*
- No *After the last question in this section, stop filling in this form.*

26. **Would you like a copy of the report when it is complete?**
Mark only one oval.

- Yes *Skip to question 27.*
- No *Stop filling out this form.*

27. **Contact**
Please provide your email here (we will only use your email adress based on answers above regarding contact/receiving a copy of finished review)

Powered by
Google Forms
