# Supplementary material for: Coral restoration – A systematic review of current methods, successes, failures and future directions
Source: PLoS One. 2020 Jan 30;15(1):e0226631. doi: 10.1371/journal.pone.0226631 (PMC6992220; doi:10.1371/journal.pone.0226631)
Supplement: S2 File — (DOCX) [file pone.0226631.s002.docx]

**S2 File – Data collected from each case study**

| **Collected data column** | **Description** |
| --- | --- |
| ID | Unique ID for case study |
| Year | Year of Publication or project conducted for unpublished data. |
| Authors | Authors listed |
| Title | Title of record |
| Publisher / Organisation | Journal or Publishing house/organisation |
| Full reference | Full reference |
| Publication Type |  |
| Public URL |  |
| Coral Restoration method | As defined in Table 1 |
| Country |  |
| Location |  |
| For geocoding |  |
| Latitude |  |
| Longitude |  |
| Disturbance | Was the restoration effort in DIRECT response to a specific disturbance i.e. COTS, storms, bleaching etc? |
| Objective | Based on Hein et al 2017 and also objectives as developed in survey questionnaire. |
| Tourism | Was there an explicit objective to enhance tourism? YES/NO |
| Stewardship? | Was there an intentional objective to increase local stewardship/ownership of coral reefs? 'the job of supervising or taking care of something' or 'the careful and responsible management of something entrusted to one's care' |
| Method specifics |  |
| Method of attachments (coral fragments) |  |
| Novelty in method | What does this study add to the knowledge of coral restoration? (as reported by authors) |
| Spatial Scale | Extent of area attempted to be restored (m2). If nursery phase only: not relevant |
| On going maintenance implemented | YES/NO |
| Location rationale | Why was location selected? |
| Coral Species |  |
| Genera |  |
| Morphology | Branching/massive/foliose/encrusting/tabular/Columnar/Soft/Mixed |
| Single species? | YES/NO |
| species rationale | Why was this species selected |
| Source of coral fragments | transplantation - fragments = from nearby reef  transplantation - colonies = from nearby reef  translocation - fragments = from reef some distance away  translocation - colonies = from reef some distance away  nursery - transplant = fragments grown in nursery, but sourced wild  nursery - self-sustaining = fragments grown in nursery from 'mother colony'  corals of opportunity= already broken fragments collected from a reef  sexual reproduction - coral planula caught during spawning events  recruitment - natural recruitment without intervention |
| Controls? | If control is needed (i.e. the authors are making statements that can not be substantiated without a control): yes/no  otherwise: none needed |
| Baseline Monitoring? | YES/NO |
| Post Monitoring? | YES/NO |
| Post monitoring length (months) |  |
| Biological/Ecological? | What were the monitoring objectives?  Biological = measuring fragment growth/survival etc  ecological = measuring overall coral cover, fish abundance etc |
| Variables measured |  |
| survival % (if measured) | Average of survival reported in study |
| MAX survival | Highest survival reported in study |
| Evidence of successful restoration outcome? | Authors perspective, explicitly stated |
| Indicators of success | What variables are used to justify success? |
| Authors/Practitioners main results/conclusions |  |
| technical 'lessons learnt' |  |
| Temporal Scale |  |
| Objective = Outcome? | (YES/NO)  Were the methods tailored to (and the outcome) meet the stated primary objective? i.e.. is the stated objective to restore a functioning ecosystem, but the methods are only addressing how to make coral nubbins grow best? |
| Outcome | Biological/ecological |
