# Supplementary material for: Coral restoration – A systematic review of current methods, successes, failures and future directions
Source: PLoS One. 2020 Jan 30;15(1):e0226631. doi: 10.1371/journal.pone.0226631 (PMC6992220; doi:10.1371/journal.pone.0226631)
Supplement: S3 File — (DOCX) [file pone.0226631.s003.docx]

**S3 File -** **Detailed information about coral restoration methods, technique and success**

**Document organisation:**

Asexual propagation methods 1

Transplantation 1

Harvest 2

Transport 2

Attachment 3

Outplanting design 4

Direct transplantation 6

Coral gardening 8

Nursery phase 9

Transplantation phase 11

Genetic diversity in coral gardening 13

Micro-fragmentation 14

Sexual propagation methods 16

Larval enhancement 16

Substratum enhancements 18

Artificial reefs 18

Substratum stabilisation 21

Substratum enhancement with electricity 22

# Asexual propagation methods

## Transplantation

The earliest developed and most common method of coral restoration (used in 70% of reviewed projects) involves transplantation of coral fragments, which, in essence, could be seen as a simulation of asexual reproduction through fragmentation [1,2]. This technique is also called ‘asexual propagation’ or ‘fragmentation’. The method bypasses the post-settlement demographic bottleneck of coral larvae, characterised by high mortality and slow growth, common in the life-histories of many hard-coral species [3-6]. Since pioneering fragmentation experiments to measure coral growth in the early 20^th^ century [7], the technique has evolved into multiple interventions specifically aimed at coral restoration. Options that have been explored include direct transplantation, the use of an “intermediate” nursery phase (also described as “coral gardening”), and micro-fragmentation. Regardless of the methods employed, interventions with coral transplantation have three steps in common, 1) the collection of corals, 2) transport, and 3) transplantation (also often called “outplanting”) onto the restoration site. Below we describe the general methods shared by each transplantation technique in terms of harvest, transport, attachment and outplanting design, and then explore details about each specific intervention type.

### Harvest

The primary method of sourcing corals is by harvesting fragments from nearby donor reefs (45% of case studies). Fragments are often removed from donor corals using surgical bone cutters, wire cutters [8] or simply by hammer and chisel. While larger fragments generally have higher survival rates [e.g. 9], collecting large fragments can be detrimental to donor colonies. It is commonly accepted that harvesting 10% or less of live tissue from donor colonies prevents significant sub-lethal effects [e.g. 2,10]. In 22% of case studies, a non-destructive method of coral collection was used, whereby fragments that are already detached from corals are collected (‘corals of opportunity’, [online figure](https://public.tableau.com/views/CoralRestorationDatabase-Visualisation/Coralrestorationmethods?:embed=y&:display_count=yes&publish=yes&:showVizHome=no#1), [11-13]. These coral fragments were dislodged either through natural processes such as wave action, fish activity or by mechanical disturbances such as ship groundings.

### Transport

A wide variety of methods have been used to transport coral fragments from the donor to the restoration sites, and largely depend on the conditions at each site and the distance involved. If the restoration site is close by, fragments can be transported underwater in crates or bins by scuba divers ([e.g. 3,14], but if the site is further away, transport by boat may be necessary. While it is generally recommended to reduce exposure to air and sunlight by transporting fragments in seawater and providing shade [8], responses to transport method vary between species. For example, Kaly [15] found that while *S. pistillata* and the gorgonian *Rumphella* sp*.* responded negatively to exposure to air during transport (two hours under a wet tarpaulin), *Acropora gemmifera* and *Favia stelligera* did not show an equivalent negative response. Similarly, Harriott and Fisk [16] reported no significant differences in survival between corals transported in water and those exposed to air for less than an hour. However, when exposure exceeded two hours, survival rates dropped significantly.

### Attachment

Once corals are at the restoration site, it is generally accepted that attaching fragments to hard substrata results in higher survival than merely placing them onto the seabed [e.g 17,18]. Being firmly attached to the substratum allows for coral tissue overgrowth and attachment to the benthos, while even slight movement of fragments can prevent attachment [e.g. 15]. Survival of loose fragments may also depend on the substratum type. For example, in an experiment testing different attachment methods, no fragments survived when scattered on sand, while approximately 80% of fragments scattered on coral rubble survived [19]. The most common method of attaching corals was with epoxy (30%), primarily in the form of a putty that hardens when two components are mixed underwater. Other common attachment methods included cable ties (i.e. ‘zip ties’ 20%) and cement (10%). Cable ties can be attached to nails or stakes driven into the substratum or dead corals present on the restoration sites [20-22]. Underwater cement can be premixed and squirted into cracks and crevices where fragments can be lodged [23,24]. Alternatively, cement can be cast into disks *ex-situ* with the base of coral fragments encased in the cement, or attached with glue [8,e.g. 25,26]. Ultimately, providing that fragments are attached in a way that does not permit movement, there appear to be negligible differences in growth or mortality between different types of attachment [18,24]. This is reflected in our data as well, where the average survival of corals between the most common attachment methods was between 60-70% ([online figure](https://public.tableau.com/views/CoralRestorationDatabase-Visualisation/Coralrestorationmethods?:embed=y&:display_count=yes&publish=yes&:showVizHome=no#1)). This suggests that, in terms of growth and survival, the method of attachment can be tailored to what is available and suitable for the reef site and project. However, these methods differ markedly in terms of time and labour costs (i.e. scattering fragments is substantially faster than gluing fragments to precast cement pucks). Finally, it should be noted that attachment methodologies need to be suited to the species to be restored and wave energy at the restoration site. Slow growing species in high energy environments require the most secure attachment, while fast growing species in low wave energy environments will need the least.

### Outplanting design

Finally, the density, pattern and species composition planted onto the restoration site can also affect the survival and growth of transplanted corals. However, perhaps due to the paucity of studies that have explored these factors experimentally, most such considerations appear to be species-specific. For example, when transplanting corals in monospecific versus mixed-species groups, Cabaitan et al., [27] demonstrated that species composition affected survival for some corals (*P. frondifera*), but not others (*P. cylindrica)*. Similarly, while some studies report that the highest growth and survival is achieved when corals are transplanted in the same orientation as they were growing in when harvested [14,28,29], others report no effect of transplant orientation on the survival of coral fragments [17,30]. Recent studies have explored how the density and arrangement of coral fragments can influence their growth and survival, and reef biodiversity [31]. For example, Ladd et al. [32] demonstrated that fragments of *A. cervicornis* tend to grow faster and lose less live tissue at moderate transplantation densities (3 corals m^-2^) than when planted closer together. However, the relationship between transplantation density and the health of transplants was not linear, with lower density groups (0.75 and 1.5 corals m^-2^) exhibiting slower growth than intermediate density groups. Similarly, Griffin et al. [33] demonstrated that *A. cervicornis* fragments tended to grow more vertically in larger groups, presumably due to increased competition for light and food with neighbouring fragments. In support of the intermediate densities for outplanting, some practitioners have found that predation by fire-worms and corallivorous snails increased when colonies were outplanted close together (Case Study 4, [8]). These experiments were conducted using a single branching coral species (*A. cervicornis*), and recommendations may vary with other species or growth morphologies. Indeed, Shaish et al. [34] found no difference in survival or growth of *Montipora digitata* fragments transplanted 10 or 20 cm apart.

## Direct transplantation

Direct transplantation is one of the earliest coral restoration methods to be developed [e.g. 1,35], and involves the harvesting of corals (fragments or whole colonies) at a donor site for transplantation at a recipient restoration site, without an intermediate nursery phase. There are 94 descriptions of direct transplantation in the review database, representing 20% of all records. This intervention was more common in the peer-reviewed literature, with only six records in the grey literature, and none from the survey. Three quarters of case studies of direct transplantation harvested fragments (59%) or whole colonies from nearby reefs (15%). This method assumes that the donor reef can withstand harvesting [36] and that the receiving degraded reef is subject to conditions that are favourable for coral growth and reef establishment [37]. While the harvesting of whole colonies may be less sustainable than harvesting of fragments, it is most common in programs aimed at salvaging corals from planned construction activities that would otherwise kill them [e.g. biodiversity offsets 38-47].

The success of direct transplantation depends on the size and health of the fragments, the method of transportation and attachment (refer to general description of harvest and attachment above), and other extrinsic factors such as environmental conditions in the months following the transplantation, when coral fragments are stressed and vulnerable [48]. Overall, direct transplantation studies reported an average survival of 64%, with 20% reporting >90% survival of transplanted corals ([online figure](https://public.tableau.com/views/CoralRestorationDatabase-Visualisation/Coralrestorationmethods?:embed=y&:display_count=yes&publish=yes&:showVizHome=no#1)). Direct transplantation has primarily involved fast-growing corals, with more than half of case studies using branching coral morphologies. Fish abundance, biomass and diversity can increase rapidly when compared to denuded control reefs [31,49-51], but very few studies have monitored the longer-term results of direct transplantation on coral reef communities beyond the survival and growth of the fragments themselves. Fish and invertebrate communities can develop in density and species richness to mimic undisturbed reef in a relatively short time [52]; coral reef fishes can respond rapidly to the change in benthic composition and rugosity at a restored reef [50,51,53]. Coral colonies relocated from dredging or construction areas may thrive in a suitable new location [46,47]. However, the paucity of studies that have monitored restored corals for longer than 12 months highlights the need for caution when generalising the potential to re-create viable coral populations and communities.

Among peer-reviewed studies that reported the results of direct transplantation, 71% reported a successful outcome based on the parameters measured by the authors (usually survival and growth). Comparatively, 100%, and 60% of survey respondents and grey literature documents, respectively, reported a successful outcome. Success, in the short term (12 months or less), was usually expressed as a survival rate of at least 50% and some measure of positive growth.

Due to the wide variety of species used in direct transplantation studies, and therefore the low replication within species, it is difficult to draw conclusions at a species level. Similar to the overall findings from the study, the genera with the highest survival tended to be those with low replication, suggesting that survival may be less species and genera specific and more related to the intervention type, and to the environmental conditions at the restoration sites. Out of the morphologies represented in the dataset, soft corals had the highest survival (survival 67%, [54,55]) followed by tabular and branching morphologies (57 and 56% respectively, [online figure](https://public.tableau.com/views/CoralRestorationDatabase-Visualisation/Coralrestorationmethods?:embed=y&:display_count=yes&publish=yes&:showVizHome=no#1)). A few studies that measured variables beyond survival and growth reported successful spawning [14], increased coral cover

[31,56], increased fish species richness and abundance [31,46,49,53,57], fish recruitment [19] and macroinvertebrate abundance [31].

Insufficient data exist for most species or genera, however *Acropora cervicornis* specifically, the Acropora genus broadly, and branching corals in general tend to achieve approximately 60-70% survival. For this reason, and based on the stoplight approach of Schopmeyer et al. [10] we suggest setting 70% survival in outplanted corals as a benchmark target of success.

The main lessons shared by direct transplantation concern which species, transport and attachment methods and receiving environments tend to yield the best results in terms of survival and growth of fragments. Transplanted fragments and colonies fare better when attached to the substratum with adhesive or tied to metal stakes or poles; unless the restoration site is in perpetually still waters, re-attachment rates of unattached fragments are too slow to withstand the effects of water movement [19,58]. Successful establishment of transplanted corals is often species-specific, and tends to work best with fast-growing species

[31,56,59]. The density of transplanted fragments and the identity of neighbouring species can also affect the survival and growth rates of direct transplants [60,61]. The seasonality of transplantation and the quality of the receiving environment were also found to be important [62,63], but this is true of any transplantation or translocation project.

## Coral gardening

Continuous harvesting of coral fragments may have detrimental effects on donor corals and populations. In response to this, a more sustainable model has been developed where coral recruits or small fragments are raised in intermediate nurseries, prior to outplanting on restoration sites. While growing in the nursery, the coral fragments are being regularly maintained (e.g. by removing any algal growth) and are relatively safe from predation, storm surges or wave energy. Coral gardening, also referred to as “coral aquaculture” or “coral farming”, is essentially mariculture of coral fragments for the purpose of coral reef restoration. The technique was modelled on silviculture, where trees are grown from seeds in nurseries, and later outplanted to restore degraded forests [64-66]. This technique is used for both commercial and reef restoration purposes. Additionally, this technique is popular in community based conservation and stewardship activities (e.g. conservation groups, NGOs and tourism operators) with a strong focus on socio-economic values[67]. The general goal of coral gardening for restoration purposes is to protect corals from damaging conditions during their most vulnerable stages, with the intention of planting them onto damaged reefs once they have reached a size threshold at which their survival post outplanting would be high [68]. In this review, 47% of case studies involved coral gardening, with a majority of records focusing on the transplantation phase of the concept (transplantation phase 23%, nursery phase 16%, both phases 8%). This technique has been extensively reviewed in prior publications, and so we briefly summarise the patterns observed in the current review. For detailed descriptions of methods and techniques we direct the reader to previous reviews [2,8] and the Coral Restoration Module of the Reef Resilience website [www.reefresilience.org](http://www.reefresilience.org/).

### Nursery phase

Corals are raised in either field-based (*in situ*), or land-based *(ex situ*) nurseries, depending on local conditions. Field-based nurseries are best placed in sheltered environments where they can be closely monitored and where conditions are favourable for the survival and growth of coral fragments. Land-based nurseries consist of aquaria or tanks where environmental conditions can be controlled. A multitude of different field-based nurseries have been designed, tailored to different environmental conditions. Coral nurseries can be in the form of structures placed on the substratum such as concrete bases, tables or frames [69,70], or mid-water structures such as ropes [9,69] or PVC ‘trees’ [e.g. 70], or dead coral bommies [71]. Fixed tables and nursery trees was the primary coral nursery method among survey respondents, while there was more diversity in nursery types in the published literature. One of the few studies that compared the merits of field-based versus land-based nurseries found little difference between the two methods. Becker and Mueller [17] explored the difference in survival and growth of *A. palmata* and *A. cervicornis* in *ex situ* and *in situ* nurseries, and found no difference in extension rates of *A. cervicornis*, but greater linear growth in *A. palmata* fragments reared in the field compared to tanks. Both species exhibited larger basal growth of fragments in tanks compared to field nurseries. One study explored the use of bamboo as material for a nursery but reported a failure of the coral fragments surviving due to disintegration of the material underwater [25].

Nurseries are stocked by removing tissue and skeleton (from a few polyps to small branches) from healthy wild coral populations, collecting “corals of opportunity” (corals fragmented through disturbance), or collecting propagules from adult colonies spawning in captivity [2]. Species considered favourable for coral gardening are those with high growth rates, rapid healing capacity, a natural tendency to use fragmentation for sexual reproduction as well as a tolerance to a range of ambient conditions [72]. For example, *A. cervicornis* is used in coral nurseries throughout the Caribbean, because, besides its historical importance for providing habitat structure and its current endangered status, it possesses these qualities [22]. Increasingly, coral nurseries are developed to be self-sustaining after the first wild collection of a ‘mother’-colony, which are used to produce subsequent generations of coral fragments (e.g. case studies in [8]), although this does raise long term issues around the genetic diversity of restored populations.

Advocates of using a coral nursery phase for reef restoration point to improved growth and survivorship rates of fragments, compared to direct transplantation; survival rates of >75% are common [69,73]. This is not echoed in our dataset, where direct transplantation studies reported 64% average survival, while coral gardening studies (i.e. those with an intermediate nursery phase) reported an average 65% survival in the outplanting phase (including studies that report only on outplant success, and those that include both stages of coral gardening). The average survival of fragments in case studies that reported survival of corals in nurseries (n=41) was 73%. However, another important factor in assessing the use of nurseries is the inherent cost and maintenance required to keep corals in a healthy condition before they are transplanted. For instance, all structures require a degree of cleaning and maintenance to prevent growing corals being overgrown or smothered by fouling organisms [74]. Recently, trials of attracting invertivorous fishes to nurseries proved successful and cost-effective [75], but studies that seek cost-cutting mechanisms are scarce.

There is a need to question whether the direct comparison of survival rates between direct transplantation and coral gardening is valid, given that coral gardening experiences mortality rates at each stage (i.e. nursery and outplanting). The ultimate survival of fragments may be more accurately expressed as a proportion of nursery survivors. This is particularly true in nurseries that are not self-sustaining (i.e. rely on harvesting fragments from the reef for each generation of outplants). Accordingly, we calculated the actual survival for fragments from case studies by expressing the average outplant survival (66%) as a proportion of the average nursery survival (73%). This reveals that the true overall survival of corals in these case studies could be as low as 48%. While this calculation may not be valid for all case studies in this review, it does highlight the challenge in comparing survival rates between intervention types, and the need for standardisation in reporting outcomes.

### Transplantation phase

Ideally, nursery-reared corals are transplanted (or ‘‘outplanted’’) from nurseries to reef restoration sites to bridge spatial gaps between existing populations, enhance local coral abundance, supplement genetic and genotypic diversity, promote natural recovery through the restoration of sexually reproductive populations, and create habitat structure for the colonisation of sessile and mobile reef organisms [76].

Survival of nursery-reared corals transplanted to restoration sites is dependent on a number of factors, including the size of fragments, their genotype and health, the season of outplanting, techniques used to secure colonies to the reef, the physical environment of the restoration site, the presence and abundance of coral predators, and the substratum type and benthic community (see also “Direct transplantation” above). For instance, macroalgal cover in the receiving environment can overgrow outplanted colonies [48], and sediment can smother fragments and impede growth after transplantation [77]. In the absence of disturbance, a review of coral restoration studies using *A. cervicornis* in the Caribbean reported high survival rates of transplanted corals within the first two years [10]. Before outplanting, nursery-grown corals have to be resilient and large enough to sustain themselves without the need of any further human intervention.

Similar to direct transplantation studies, the success of transplanted corals in creating viable coral populations is usually only monitored for a short time. Fifty percent of all studies monitored restored coral populations for 12 months or less, and very few of these studies recorded anything other than biological variables such as survival and growth of transplanted corals. Survival rates need to measured in the context of the proportion of fragments or colonies surviving the nursery stage (see above). Thus, it appears that the emphasis of coral gardening is still on optimising nursery techniques and ensuring survival in the first year of outplanting, rather than on following the results of outplanting towards the creation of a viable coral community that enhances or restores the overall reef system.

There is a consensus among coral gardening practitioners that corals reared in nurseries are best allowed to reach a certain size before outplanting, and that this size may vary between species [78]. Many practitioners focused their discussion about “lessons learned” on the cost and effort of coral gardening. Others gave details on the necessary maintenance of corals in nurseries, and how to address problems such as algal overgrowth and dislodgement by predatory fishes. When ecological variables were monitored, there was generally an increase in coral cover [28], fish abundance [31,79] and natural coral recruitment [79]. In areas where corals or coral fragments had been outplanted, ongoing success was most often hampered by natural disturbances, such as COTS outbreaks [79].

In contrast to ex-situ nurseries, which are isolated and relatively sterile, in situ coral nurseries and transplantation sites are open to recruitment of reef organisms, including fishes and invertebrates that may harm young corals [75,80]. Whilst in some cases fishes may inadvertently clean algae and biofouling organisms from around the growing corals, they may also prey on the corals, damage them during grazing [80], or, in the case of territorial damselfishes, grow algae that can smother or compete with the corals [81]. Further, there is ample anecdotal evidence that reef fishes like parrotfish target vulnerable outplants over existing coral colonies of the same species. Similarly, multiple case studies mentioned predation by *Drupella* snails and other gastropods on both outplanted corals and *in situ* nurseries [24,82-85], suggesting that these corallivores are attracted to corals disturbed during interventions. Site specific conditions should therefore be considered during the planning phase of restoration projects.

### Genetic diversity in coral gardening

If the goals of restoration are to include resilience to existing or future stresses, the consideration of genetic diversity is crucial [86]. Acroporids, which are used preferentially in coral gardening, naturally reproduce asexually through fragmentation, so the recommended genetic diversity ratio reflects the proportion of unique genotypes per number of colonies sampled in a specific stand or thicket [86]. The clonal processes preferentially used in coral gardening inherently limit resilience; assisted fertilization [87] or creating nursery stocks from the larvae of brooding corals [88] could be valuable tools for maintaining genetic diversity in coral gardening. The NOAA recovery plan

[89] suggests a target genetic diversity ratio of 0.5 for both *A. cervicornis* and *A. palmata* [90]*.*

Three peer-reviewed studies identified in this review have specifically tested the viability or performance of different genets of the same species in a coral gardening or transplantation context, especially for the purposes of recommending stronger genets for transplantation (e.g. Ross 2014). In *A. cervicornis*, it is understood that there are strong differences in growth rate and susceptibility to temperature, fouling and abrasion stresses between genets (Ross 2014, Ladd et al. 2017); there can be up to a six-fold difference in relative growth based on genotype alone (Bowden-Kerby at al. 2008). There are also differences in thermal tolerance, which may become one of the most important factors in a warming ocean (Ladd et al. 2017). Differences between genets that are apparent in the nursery may be less obvious in outplanted corals [91], as characteristics of the receiving environment may have overriding effects on survival and growth [92]. Furthermore, genotypes considered poor or strong survivors or growers may not perform consistently in different locations or years, and should not be dismissed [93].

Acroporids are broadcast, hermaphrodite spawners, and cross-fertilization can be limited by distance between colonies and populations; multiple genets of each species are ideally placed in proximity to each other to facilitate heterozygosity in mass spawning events [72,94]. It is also important to consider that the genotypic composition of restored coral populations is at least as important as genotypic diversity for restoration success [95]. The effects of different levels of genetic diversity on the long-term persistence and function of a restored reef remains to be explored.

## Micro-fragmentation

Less than 5% of transplantation studies have been conducted with slow growing life histories. Massive corals have largely been overlooked, mainly due to their slow growth and thicker skeletons, which are less amenable to fragmenting [96]. However, recent research from Mote Marine Laboratory, based on decades of aquarist experimentation, has developed a ‘micro-fragmentation’ technique that enables massive and encrusting corals to be mass-produced and outplanted using concepts developed for coral gardening [96,97].

A diamond blade saw is used to cut small fragments (1 cm^2^) of massive corals, which are then mounted on tiles. The tiles are kept in artificially lit and aerated aquaria for several weeks, after which they are placed in large outdoor flow-through aquaria. After approximately 12 months, the fragments can either be further sub-divided to generate new micro-fragments or outplanted. Micro-fragments that are secured to reef substrates or dead coral bommies in an array will readily fuse together to form a larger colony (i.e. ‘re-skinning’). The technique has been tested on ten massive coral species, with emphasis on three species determined to be most suitable for large-scale field trials (*Montastrea cavernosa*, *Orbicella faveolata* and *Diploria clivosa*; [96]). The research outcomes show high survival and rapid growth of fragments (>99% survival, [96,97]. Recent research has shown that re-skinned corals reached sexual maturity more rapidly (~18 months) than naturally growing corals (~10 years) (T. Vardi *pers. comms*).

To date, no study has reported results of micro-fragmentation outside controlled aquarium environments. However, some are beginning to test the effects of fragment size and different environmental conditions on fragment health. Hall et al. [98] found that pH interacts with fragment size to affect physiology and recovery from lesions in a species-specific way. Forsman et al. [97] tested growth and survival of fragments in two tanks; one cleaned and maintained tank, and one established mesocosm tank that contained other corals, reef organisms and fishes. They reported faster growth but lower survival of fragments in the established tank, suggesting that results may vary when tested in a natural reef environment, however these results require further replication.

Similar to coral gardening, and many active interventions currently in use, micro-fragmentation relies on an intermediate phase of *ex situ* nursery rearing. Intuitively, substantive cost reduction and efficiency increases could be gained by forgoing the intermediate nursery phase, but this could also lead to decreased survivorship. This could be an important avenue for future research, as no current published literature exists on benefits of nursery rearing versus direct transplantation on the reef for micro-fragmentation studies.

# Sexual propagation methods

## Larval enhancement

The process of larval enhancement (also known as ‘larval propagation’, ‘sexual propagation’ or ‘larval re-seeding’) aims at increasing the rates of larval production and settlement leading to increased recruitment success. These methods are designed to overcome the natural demographic bottlenecks where coral fertilization rates may be limited on reefs with low coral cover and asynchronous spawning, and planktonic development of embryos and larvae may result in a high proportion of coral larvae being swept away from reefs and therefore failing to settle or recruit [99-101]. As such, the methods are mainly aimed at enhancing larval settlement and recruitment at sites that have experienced recruitment failure, or drastically reduced recruitment levels. Sexual reproduction methods have advantages over asexual propagation methods because they increase genetic diversity among restored coral populations, thereby enabling increase rates of adaptation and improved resilience [102]. Larval enhancement also has potential for increased scales of restoration on degraded reefs.

There are two main types of larval enhancement strategies to enhance recruitment. First, interventions can collect or rear embryos and larvae to increase settlement on artificial or engineered structures that are later placed on reefs. For example, work in Japan showed that culture of spawned coral gametes can provide access to large numbers of embryos and larvae [103], some of which can be induced to settle on settlement surfaces that can be outplanted onto reefs [104,105]. Settlement of *ex situ* cultured larvae and transplantation of settled juvenile corals onto reef areas has also been successfully trialled in the Philippines, with increased survival rates evident among larger and older age classes of recruits when outplanted onto reefs [106-109]. Recently, Chamberland et al. [110] ‘seeded’ concrete tetrapods, with Caribbean *Favia fragum* larvae that had been fertilised and reared *ex situ.* The ‘seeding units’ were scattered onto a degraded reef area, after a four-week juvenile coral rearing period. Approximately 10% of settled larvae survived, and 56% of seeding units harboured at least one *F. fragum* individual after one year. The authors concluded that the main advantage of this method over others is the speed of outplanting compared to methods which attach coral fragments individually.

Another larval-based method was used by Edwards et al [111] in Palau where *A. digitifera* larvae were reared *ex situ* and then allowed to settle on 1.2 x 0.9 m concrete pallet balls and settlement tiles enclosed in a tent. Although the authors recorded high initial larval settlement rates on tiles, there were no significant differences in the mean number of coral recruits surviving on larval enhanced versus control pallet balls after 13 months. The authors speculated that this may be due to high post-settlement mortality combined with high rates of natural coral recruitment in the healthy reef area used for the study [111].

In a second larval enhancement technique, coral gametes are collected during spawning, embryos and larvae are reared in holding tanks or on the reef, and then larvae are released directly onto the reef in enclosures that retain them during the settlement period [6,112,113]. Heyward et al. [112] collected spawned acroporid gametes and reared embryos and larvae in small 1.8 m floating ponds for six days at Ningaloo Reef, Western Australia. When larvae were competent to settle, they were funnelled into a floorless mesh tent, anchored to the reef substrata, and pumped onto an enclosed 1.8 x 1m reef area for 20 minutes, and then the tent was moved to an adjacent area for 12 hours. Although the numbers of larvae pumped onto the experimental reef plots and initial settlement rates on tiles were not quantified, recruitment rates on tiles in the larval enhancement plots monitored after six weeks were up to 100-fold higher compared to control sites. However, this study was not conducted with a coral restoration objective, so no ongoing monitoring was undertaken, and therefore the longer-term juvenile survival and restoration outcomes are not known.

More recently, longer-term replicated larval enhancement and recruitment trials have been completed successfully on highly degraded reef areas in Northern Luzon, Philippines [6,114]. They demonstrated that mass larval settlement on degraded reef areas (4 x 6 m) can significantly enhance recruitment and re-establish a breeding population of A. *tenuis* colonies after three years. Spawned gametes were collected from thirty gravid colonies, and embryos and larvae were reared in *ex situ* tanks for four days, then they were transferred onto replicate reef plots and retained in low-cost fine mesh larval enclosures for five days. High rates of larval settlement were recorded on settlement tiles in the larval enhancement plots whereas none settled on control tiles. As expected for broadcast spawning marine invertebrates, mortality rates of settled corals were highest in the first five months after settlement, after which survivorship stabilised as recruits reached visible size Rapid growth lead to early onset of sexual reproduction in many colonies and these corals are now dominating the larval enhancement plots. Further, they have spawned annually over the past three years, thereby contributing to larval production on these reefs (de la Cuz and Harrison, unpubl. data). Additional larval enhancement reef trials with larvae from other *Acropora* species and brain corals have resulted in similar patterns of settlement, recruitment and growth on other reef areas in the Philippines (Harrison et al. unpubl. data). Larger scale larval enhancement trials using 100 m^2^ reef patches enclosed in floating mesh curtains resulted in successful larval settlement on reef patches in the southern Great Barrier Reef during 2017 and long-term monitoring of recruitment, survival and growth is underway (Harrison et al., unpubl. data).

# Substratum enhancements

## Artificial reefs

About one fifth of projects (19%) described in the review involve the creation of substratum, such as artificial reefs. The creation of substratum involves structures that are placed on the seabed deliberately, sometimes to mimic characteristics of a natural reef, or for the purpose of increasing potential habitat for reef assemblages, fisheries yield and production, recreational diving opportunities and the prevention of trawling. Given the search parameters of this systematic review, we have only included artificial reefs with an explicit coral restoration objective, so projects with other objectives are likely underrepresented. The structures are usually placed on, or attached to, substratum that has been damaged (e.g. ship grounding sites) or that is otherwise unsuitable for coral settlement (e.g. loose rubble or soft sediment). In many cases, artificial reefs are deployed in conjunction with other methods, such as coral transplantation. Recently, the effects of artificial reefs on coral reef communities has also been studied on existing structures such as breakwaters, groynes, and jetties [115], which can yield important information for the creation of substratum for reef restoration.

A number of materials have been tested since the onset of artificial reef creation, but the most favoured reef material is concrete, usually in the form of cubes, blocks and pipes [116]. Other materials used, in order of decreasing preference, are gabbro, granite, sandstone, and terra-cotta [117]. Increasingly, engineered structures (e.g. EcoReefs, BioRock, ReefBalls) are designed with greater 3-dimensional complexity, in attempts to mimic coral reef habitats more closely (e.g. [www.reefball.com](http://www.reefball.com)). It is generally understood that the choice of material will influence the success of coral settlement, and hence the development of the entire benthic community (Burt et al. 2009). Additionally, the complexity of artificial reef structures will affect the settlement of benthic organisms [117,118]. Despite the large number of artificial reefs deployed worldwide for a variety of reasons, very few studies have monitored their development beyond the early stages of colonisation by benthic organisms [119]. This may be an artefact of our focus on artificial reefs developed for coral restoration specifically, contrasted to those developed for fishery augmentation.

In the past decade, Mars Incorporated have developed a modular approach to restoring corals, particularly suitable to deploy on unstable substrate. The technique uses small, modular, open structures consisting of three steel bars covered with several layers of anti-oxidation coating and a mixture of sand and calcium carbonate cover. The structures resemble a 6-legged spider, each covering approximately 1m^2^ and 30 cm high. Corals of opportunity are tied to the ‘spider’ structure with cable ties, after which the spiders are deployed to degraded reefs. The units are tied together, creating a structure more likely to withstand storms. On unstable substrates, a mesh is deployed underneath to stabilise the rubble. According to developers, the spiders require maintenance in the first five months to remove algae after which they can sustain themselves without further intervention (Presentation on 17/08/2018 at Reef Restoration Workshop, Bali, Noel Janetski, Mars Inc.). The organisation further states that the spiders are very effective and can restore a reef with a cover of up to 70% after only 3 years. The technique has been employed since 2007, and has been used at larger scales in Indonesia where reefs are affected by dynamite fishing and cyanide fishing [120]. The structures used in this process are presumably incorporated into the reef structure over time, however the longer-term effectiveness, impact and stability of this modular system remain to be evaluated.

Recently, interest has increased in using artificial reefs to provide shoreline protection, in particular as insurance payouts for coastal damages from storms have risen in the past decade [121]. For example, gabion baskets were deployed to provide a breakwater to reduce erosion and flooding in Grenville Bay, Grenada. The gabions were supplemented with transplanted corals from the local areas to replace reefs lost from hurricanes, and early results suggest the artificial reef substantially reduces the wave energy reaching the shore [122].

The success with which an artificial reef functions as a complete ecosystem will require long-term comparisons between artificial reefs and natural reefs subject to the same environmental conditions [119,123]. A study that compared artificial reefs and nearby natural reefs for five years found that at the end of this period, the similarity of the scleractinian and octocoral community composition was 70% and 63%, respectively[119]. Further, a study that monitored the development of coral reef communities on two types of artificial reefs and compared them with nearby natural reefs found that it took four years for the benthic and fish assemblages on the artificial reefs to stabilise, highlighting the need for such long-term studies to assess the success of this method of habitat creation [124]. Blakeway et al. [125] found that colonisation of an artificial reef in turbid waters by natural recruitment resulted in significant numbers of recruits but that after six years the cover on the artificial reef (2.4%) was approximately a tenth of that on co-occurring natural reefs. The process of colonisation may therefore be a slow process on turbid inshore reefs.

## Substratum stabilisation

The direct physical restoration of damaged substratum mostly involves stabilising rubble over an area that has been affected by storms or ship groundings. The rationale is that corals settling onto a damaged reef cannot successfully recruit to loose substratum, as survival rates are low [61]. While substratum stabilisation has been used relatively often in US territorial waters, funded by insurance claims following ship-strikes [126], there is a paucity of published literature that clearly describes methods and techniques (4% of case studies in this review). The most common method is to install mesh or netting over the rubble to prevent further movement. This is generally a precursor to the transplantation of corals onto the damaged area [61]and/or the additional deployment of artificial structures. Other methods include metal spikes driven into loose substratum [127], rock piles on unstable degraded reef areas [127], and open cement structures placed to contain loose substratum [3,128].

Only four published studies and one unpublished report describing substratum stabilisation were available, preventing analyses of general trends or outcomes. Substratum stabilisation was mentioned as a method of preparing for coral transplantation in a number of studies, but the transplantation was the focus of the study, and the effects of stabilisation were not assessed. One study that evaluated a range of different stabilisation mechanisms found that hard substrata were more successful in attracting coral recruits than soft structures such as nets, and that the recruits also survived better on hard substratum [127].

In addition to stabilising substrates, the use of artificial structures may avoid issues with unstable substrates. For example, the six-legged artificial reef structures (‘spiders’) described above circumvent the problem of unstable and soft substrates, by elevating transplanted corals above the substrate.

## Substratum enhancement with electricity

In five percent of substratum enhancement studies, electricity was used in an attempt to encourage faster growth and higher survival of coral transplants. This concept and technique was pioneered by Wolf Hilbertz in the 1970s and developed into a commercial product (Biorock). The aim of the technique is to mimic the chemical and physical properties of reef limestone, by encouraging the precipitation of calcium and magnesium on artificial substrates [129]. A direct electrical current is established between electrodes, and calcium carbonate and magnesium hydroxide precipitates at the cathode, while oxygen and chlorine are produced at the anode [130]. The purpose of this mineral accretion is to potentially increase calcification of coral polyps, and therefore boost colony growth and resilience to stressors. The authors and patent holders [130,131] have published books and reports on the apparent effectiveness of this technique, suggesting that it increases the growth rates, survival, stress resistance, and physiology of corals [132]. However, the technique has been controversial and experiments attempting to verify its effectiveness have had varied outcomes.

Sabater and Yap [133] described increased growth and attachment in *P. cylindrica* fragments when connected to a setup similar to that described by Goreau and Hilbertz [131]. A range of other studies have described increased survival of fragments on mineral accretion frames [134-138]. However, multiple experiments have failed to describe similar positive effects of exposing coral fragments to an electrical field. For example, Romatzki [139] found that *A. pulchra* and *A. yongei* coral fragments exposed to similar strength electrical currents as those described by previous researchers grew slower than control colonies. Similarly, Borell [140] described negative effects on growth of one species of coral (*A. yongei*) but positive effects on another (*A. pulchra*) growing on a cathode, suggesting that results may vary even between congeneric coral species. The disagreement between studies prohibits clear conclusions about the mineral accretions method. However, some studies indicate that coral fragments exposed to an electrical field will attach more rapidly to frames and structures [133-135,137-141], which could increase survival to some degree.

1. Maragos JE. Coral transplantation: a method to create, preserve and manage coral reefs. Sea Grant Advising Report SEA-GRANT-AR.74-03-COR-MAR-14. University of Hawaii, Honolulu; 1974 p. 30.

2. Edwards A, Gomez E. Reef restoration: concepts & guidelines. St Lucia, Australia: Coral Reef Targeted Research & Capacity Building for Management Programme iv. 2007.

3. Clark S, Edwards AJ. Coral transplantation as an aid to reef rehabilitation: evaluation of a case study in the Maldive Islands. Coral Reefs. Springer-Verlag; 1995;14: 201–213. doi:10.1007/BF00334342

4. Lindahl U. Low-Tech Rehabilitation of Degraded Coral Reefs through Transplantation of Staghorn Corals. Ambio. Allen Press on behalf of Royal Swedish Academy of Sciences; 1998;27: 645–650.

5. Zimmer B. Coral reef restoration: an overview. Coral reef restoration handbook. Taylor and Francis Boca Raton, FL; 2006;: 39–59.

6. Cruz dela DW, Harrison PL. Enhanced larval supply and recruitment can replenish reef corals on degraded reefs. Sci Rep. Springer US; 2017;7: 1–13.

7. Vaughan TW. Growth rate of the Florida and Bahamian shoal-water corals. Carnegie Institute of Washington Year Book; 1916. pp. 221–231.

8. Johnson ME, Lustic C, Bartels E, Baums IB, Gilliam DS, Larson EA, et al. Caribbean Acropora restoration guide: best practices for propagation and population enhancement. 2011.

9. Bowden-Kerby A. Low-tech coral reef restoration methods modeled after natural fragmentation processes. B Mar Sci. University of Miami-Rosenstiel School of Marine and Atmospheric Science; 2001;69: 915–931.

10. Schopmeyer SA, Lirman D, Bartels E, Gilliam DS, Goergen EA, Griffin SP, et al. Regional restoration benchmarks for Acropora cervicornis. Coral Reefs. Springer Berlin Heidelberg; 2017;276: 1–11. doi:10.1007/s00338-017-1596-3

11. Schuhmacher H, Van Treeck P, Eisinger M, Paster M. Transplantation of coral fragments from ship groundings on electrochemically formed reef structures. 2002. pp. 983–990.

12. Bruckner A, Bruckner R. Condition of restored Acropora palmata fragments off Mona Island, Puerto Rico, 2 years after the Fortuna Reefer ship grounding. Coral Reefs. Springer-Verlag; 2001;20: 235–243. doi:10.1007/s003380100164

13. Monty JA, Gilliam DS, Banks K, Stout DK, Dodge RE. Coral of opportunity survivorship and the use of coral nurseries in coral reef restoration. 2006.

14. Okubo N, Taniguchi H, Motokawa T. Successful methods for transplanting fragments of *Acropora formosa* and *Acropora hyacinthus*. Coral Reefs. 2005;24: 333–342.

15. Kaly UL. Experimental test of the effects of methods of attachment and handling on the rapid transplantation of corals. Townsville, Australia: CRC Reef Research Centre, James Cook University; 1995 pp. 1–24.

16. Harriott VJ, Fisk DA. Accelerated regeneration of hard corals: a manual for coral reef users and managers. Great Barrier Reef Marine Park Authority; 1995.

17. Becker LC, Mueller E. The culture, transplantation, and storage of *Montastraea faveolata*, *Acropora cervicornis*, and *A. palmata*: what we learned so far. Bull Mar Sci. 2001;69: 881–896.

18. Forrester GE, Rodwell CO, Baily P, Forrester LM, Giovannini S, Harmon L, et al. Evaluating Methods for Transplanting Endangered Elkhorn Corals in the Virgin Islands. Restoration Ecology. Wiley/Blackwell (10.1111); 2011;19: 299–306. doi:10.1111/j.1526-100X.2010.00664.x

19. Bowden-Kerby A. Coral transplantation in sheltered habitats using unattached fragments and cultured colonies. Smithsonian Tropical Research Institute: Panama; 1997. pp. 2063–2068.

20. Ross AM. The Decline and Restoration of Acropora Cervicornis in Montego Bay: Exploring the Anthozoics and Anthozoculture of A. Cervicornis. The University of the West Indies; 2012.

21. Hernández-Delgado EA, Montañez-Acuña A, Otaño-Cruz A, Suleimán-Ramos SE. Bomb-cratered coral reefs in Puerto Rico, the untold story about a novel habitat: from reef destruction to community-based ecological rehabilitation. Rev Biol Trop. 2014;62: 350–367.

22. Lirman D, Schopmeyer S, Galvan V, Drury C, Baker AC, Baums IB. Growth Dynamics of the Threatened Caribbean Staghorn Coral Acropora cervicornis: Influence of Host Genotype, Symbiont Identity, Colony Size, and Environmental Setting. PLoS ONE. Public Library of Science; 2014;9: e107253. doi:10.1371/journal.pone.0107253

23. Alcala AC, Alcala LC, Gomez ED, Cowan ME, Yap HT. Growth of certain corals, molluscs and fish in artificial reefs in the Philippines. 1982. pp. 215–220.

24. Dizon RM, Alasdair JE, Edgardo DG. Comparison of three types of adhesives in attaching coral transplants to clam shell substrates. Aquat Conserv. 2008;18: 1140–1148.

25. Ferse SCA. Poor Performance of Corals Transplanted onto Substrates of Short Durability. Restoration Ecology. 2010;18: 399–407. doi:10.1111/j.1526-100X.2010.00682.x

26. Bowden-Kerby A. Best practices manual for Caribbean Acropora restoration. Puntacana Ecological Foundation; 2014 p. 41.

27. Cabaitan PC, Yap HT, Gomez ED. Performance of single versus mixed coral species for transplantation to restore degraded reefs. Restoration Ecology. Wiley Periodicals, Inc; 2015;23: 349–356. doi:10.1111/rec.12205

28. Nakamura R, Ando W, Yamamoto H, Kitano M, Sato A, Nakamura M, et al. Corals mass-cultured from eggs and transplanted as juveniles to their native, remote coral reef. 2011;436: 161–168. Available: https://www.int-res.com/abstracts/meps/v436/p161-168/

29. Gomez ED, Cabaitan PC, Yap HT, Dizon RM. Can Coral Cover be Restored in the Absence of Natural Recruitment and Reef Recovery? Restoration Ecology. Wiley Periodicals, Inc; 2014;22: 142–150. doi:10.1111/rec.12041

30. Bongiorni L, Shafir S, Rinkevich B. Effects of particulate matter released by a fish farm (Eilat, Red Sea) on survival and growth of Stylophora pistillata coral nubbins. Mar Pollut Bull. 2003;46: 1120–1124.

31. Cruz dela DW, Villanueva RD, Baria MVB. Community-based, low-tech method of restoring a lost thicket of Acropora corals. ICES Journal of Marine Science. 2nd ed. 2014;71: 1866–1875. doi:10.1093/icesjms/fst228

32. Ladd MC, Shantz AA, Nedimyer K, Burkepile DE. Density Dependence Drives Habitat Production and Survivorship of Acropora cervicornis Used for Restoration on a Caribbean Coral Reef. Front Mar Sci. Frontiers; 2016;3: 105. doi:10.3389/fmars.2016.00261

33. Griffin JN, Schrack EC, Lewis KA, Baums IB, Soomdat N, Silliman BR. Density‐dependent effects on initial growth of a branching coral under restoration. Restoration Ecology. Wiley/Blackwell (10.1111); 2015;23: 197–200. doi:10.1111/rec.12173

34. Shaish L, Levy G, Katzir G, Rinkevich B. Employing a highly fragmented, weedy coral species in reef restoration. Ecological Engineering. 2010;36: 1424–1432. doi:10.1016/j.ecoleng.2010.06.022

35. Birkeland C, Randall RH, Grimm G. Three methods of coral transplantation for the purpose of reestablishing a coral community in the thermal effluent area at the Tanguisson power plant [Internet]. University of Guam, Marine Laboratory; 1979. Available: http://www.guammarinelab.org/publications/uogmltechrep60.pdf

36. Epstein N, Bak RPM, Rinkevich B. Strategies for Gardening Denuded Coral Reef Areas: The Applicability of Using Different Types of Coral Material for Reef Restoration. Restoration Ecology. 2001;9: 432–442. doi:10.1046/j.1526-100X.2001.94012.x

37. Dizon R, Yap H. Effects of coral transplantation in sites of varying distances and environmental conditions. Mar Biol. 2006;148: 933–943.

38. Plucer-Rosario GP, Randall RH. Preservation of rare coral species by transplantation: an examination of their recruitment and growth. B Mar Sci. 1987;41: 585–593.

39. Newman H, Chuan CS. Transplanting a coral reef: A Singapore community project. Coastal Management in Tropical Asia. 1994;3: 11–14.

40. Thornton SL, Dodge RE, Gilliam DS, DeVictor R, Cooke P. Success and growth of corals transplanted to cement armor mat tiles in southeast Florida: implications for reef restoration. 2000.

41. Gayle PMH, Wilson-Kelly P, Green S. Transplantation of benthic species to mitigate impacts of coastal development in Jamaica. Revista de Biología Tropical. 2005;53: 105–115.

42. Seguin F, Le Brun O, Hirst R, Al-Thary I, Dutrieux E. Large coral transplantation in Bal Haf (Yemen): an opportunity to save corals during the construction of a Liquefied Natural Gas plant using innovative techniques. 2008. pp. 1267–1270.

43. Yeemin T, Sutthacheep M, Pettongma R. Coral reef restoration projects in Thailand. Ocean Coast Manag. Elsevier; 2006;49: 562–575.

44. Kilbane D, Graham B, Mulcahy R, Onder A, Pratt M. Coral relocation for impact mitigation in Northern Qatar. Fort Lauderdale, FL, USA; 2008.

45. Kenny I, Kramer A, Wilson Kelly PW, Burbury T. Coral Relocation: A mitigation tool for dredging works in Jamaica. Cairns, Australia; 2012. p. 20A.

46. Rodgers KS, Lorance K, Richards Donà A, Stender Y, Lager C, Jokiel PL. Effectiveness of coral relocation as a mitigation strategy in Kāne`ohe Bay, Hawai`i. Reimer J, editor. PeerJ. 2017;5: e3346.

47. Kotb MMA. Coral translocation and farming as mitigation and conservation measures for coastal development in the Red Sea: Aqaba case study, Jordan. Environ Earth Sci. Springer Berlin Heidelberg; 2016;75: 439. doi:10.1007/s12665-016-5304-3

48. van Woesik R, Ripple K, Miller SL. Macroalgae reduces survival of nursery-reared Acropora corals in the Florida reef tract. Restoration Ecology. 2018;26: 563–569. doi:10.1111/rec.12590

49. Cabaitan PC, Gomez ED, Aliño PM. Effects of coral transplantation and giant clam restocking on the structure of fish communities on degraded patch reefs. J Exp Mar Bio Ecol. 2008;357: 85–98.

50. Opel AH, Cavanaugh CM, Rotjan RD, Nelson JP. The effect of coral restoration on Caribbean reef fish communities. Marine Biology. Springer Berlin Heidelberg; 2017;164: 221. doi:10.1007/s00227-017-3248-0

51. Ferse SCA. Multivariate responses of the coral reef fish community to artificial structures and coral transplants. In: Riegl BM, Dodge RE, editors. Fort Lauderdale, Florida, USA: National Coral Reef Institute, Nova Southeastern University, Dania, Florida; 2009. pp. 1230–1234.

52. Yap HT. Local changes in community diversity after coral transplantation. Mar Ecol Prog Ser. 2009;374: 33–41. doi:10.3354/meps07650

53. Ferse SCA. Artificial reef structures and coral transplantation: fish community responses and effects on coral recruitment in North Sulawesi/Indonesia. Leibniz Zentrum für Marine Tropenökologie (ZMT). Bremen, Germany: University of Bremen; 2008. p. 169.

54. Montgomery AD. The feasibility of transplanting black coral (Order Antipatharia). Hydrobiologia. 2002;471: 157–164. doi:10.1023/A:1016573926566

55. Oren U, Benayahu Y. Transplantation of juvenile corals: a new approach for enhancing colonization of artificial reefs. Marine Biology. 1997;127: 499–505. doi:10.1007/s002270050038

56. Miyazaki K, Keshavmurthy S, Biosphere SFK, 2010. Survival and growth of transplanted coral fragements in a high-latitude coral community (32 N) in Kochi, Japan. kuroshioorjp

.

57. Ferse SCA. Multivariate responses of the coral reef fish community to artificial structures and coral transplants. In: Riegl BM, Dodge RE, editors. Fort Lauderdale, Florida, USA: National Coral Reef Institute, Nova Southeastern University, Dania, Florida; 2009.

58. Guest JR, Dizon RM, Edwards AJ, Franco C, Gomez ED. How Quickly do Fragments of Coral “Self‐Attach” after Transplantation? Restoration Ecology. Wiley/Blackwell (10.1111); 2011;19: 234–242. doi:10.1111/j.1526-100X.2009.00562.x

59. Garrison VH, Ward G. Transplantation of storm-generated coral fragments to enhance Caribbean coral reefs: A successful method but not a solution. Revista de Biología Tropical. 2012;60: 59–70.

60. Raymundo LJH. Mediation of growth by conspecific neighbors and the effect of site in transplanted fragments of the coral *Porites attenuata* Nemenzo in the central Philippines. Coral Reefs. 2001;20: 263–272.

61. Lindahl U. Coral reef rehabilitation through transplantation of staghorn corals: effects of artificial stabilization and mechanical damages. Coral Reefs. 2003;22: 217–223.

62. Yap HT, Gomez ED. Growth of Acropora pulchra. II. Responses of natural and transplanted colonies to temperature and day length. Mar Biol. 1984;81: 209–215.

63. Yap HT, Gomez ED. Growth of Acropora pulchra. III. Preliminary observations on the effects of transplantation and sediment on the growth and survival of transplants. Marine Biology. 1985;87: 203–209.

64. Rinkevich B. Restoration Strategies for Coral Reefs Damaged by Recreational Activities: The Use of Sexual and Asexual Recruits. Restoration Ecology. Wiley/Blackwell (10.1111); 1995;3: 241–251. doi:10.1111/j.1526-100X.1995.tb00091.x

65. Rinkevich B. Steps towards the evaluation of coral reef restoration by using small branch fragments. Marine Biology. Springer-Verlag; 2000;136: 807–812. doi:10.1007/s002270000293

66. Rinkevich B. Stylophora pistillata: Eco-physiological aspects in the biology of a hermatypic coral. Tel-Aviv University, Tel-Aviv Google Scholar. 1982.

67. Heeger T, Cashman M, Sotto F. Coral farming as alternative livelihood, for sustainable natural resource management and coral reef rehabilitation. 1999. pp. 171–186.

68. Rinkevich B. Conservation of Coral Reefs through Active Restoration Measures: Recent Approaches and Last Decade Progress. Environ Sci Technol. 2005;39: 4333–4342.

69. Shaish L, Levy G, Gomez E, Rinkevich B. Fixed and suspended coral nurseries in the Philippines: establishing the first step in the ``gardening concept“” of reef restoration. J Exp Mar Bio Ecol. 2008;358: 86–97.

70. Nedimyer K, Gaines K, Roach S. Coral tree nursery ©: An innovative approach to growing corals in an ocean-based field nursery. AACL Bioflux. 2011;4: 442–446.

71. Cruz dela DW, Rinkevich B, Gomez ED, Yap HT. Assessing an abridged nursery phase for slow growing corals used in coral restoration. Ecological Engineering. 2015;84: 408–415. doi:10.1016/j.ecoleng.2015.09.042

72. Young CN, Schopmeyer SA, Lirman D. A Review of Reef Restoration and Coral Propagation Using the Threatened Genus Acroporain the Caribbean and Western Atlantic. B Mar Sci. University of Miami - Rosenstiel School of Marine and Atmospheric Science; 2012;88: 1075–1098. doi:10.5343/bms.2011.1143

73. Putchim L, Thongtham N, Hewett A, Chansang H. Survival and growth of Acropora spp. in mid-water nursery and after transplantation at Phi Phi Islands, Andaman Sea, Thailand. 2008. pp. 1258–1261.

74. Precht WF, Robbart M. Coral reef restoration: the rehabilitation of an ecosystem under siege. In: Precht WF, editor. Coral Reef Restoration Handbook. Boca Raton, FL, USA: CRC Press / Taylor and Francis; 2006. pp. 1–24.

75. Frias-Torres S, van de Geer C. Testing animal-assisted cleaning prior to transplantation in coral reef restoration. Costello M, editor. PeerJ. 2015;3: e1287. doi:10.7717/peerj.1287

76. Lirman D, Miller MW. Modeling and Monitoring Tools to Assess Recovery Status and Convergence Rates between Restored and Undisturbed Coral Reef Habitats. Restoration Ecology. Wiley/Blackwell (10.1111); 2003;11: 448–456. doi:10.1046/j.1526-100X.2003.rec0286.x

77. Ng CSL, Chou LM. Rearing juvenile “corals of opportunity” in in situ nurseries – A reef rehabilitation approach for sediment-impacted environments. Marine Biology Research. Taylor & Francis; 2014;10: 833–838. doi:10.1080/17451000.2013.853124

78. Raymundo LJH, Maypa AP, Luchavez MM. Coral seeding as a technology for recovering degraded coral reefs in the Philippines. Phuket Marine Biology Center Special Publication. 1999;20: 81–92.

79. Mbije NE, Spanier E, Rinkevich B. A first endeavour in restoring denuded, post-bleached reefs in Tanzania. Estuar Coast Shelf S. 2013;128: 41–51. doi:10.1016/j.ecss.2013.04.021

80. Horoszowski-Fridman YB, Brêthes J-C, Rahmani N, Rinkevich B. Marine silviculture: Incorporating ecosystem engineering properties into reef restoration acts. Ecological Engineering. Elsevier; 2015;82: 201–213. doi:10.1016/j.ecoleng.2015.04.104

81. Williams SL, Sur C, Janetski N, Hollarsmith JA, Rapi S, Barron L, et al. Large-scale coral reef rehabilitation after blast fishing in Indonesia. Restoration Ecology. Wiley Online Library; 2019;27: 447–456.

82. Clark S, Edwards AJ. Use of artificial reef structures to rehabilitate reef flats degraded by coral mining in the Maldives. B Mar Sci. 1994;55: 724–744.

83. Van Treeck P, Schuhmacher H. Initial survival of coral nubbins transplanted by a new coral transplantation technology - options for reef rehabilitation. Mar Ecol Prog Ser. 1997;150: 287–292.

84. Shafir S, Van Rijn J, Rinkevich B. Coral nubbins as source material for coral biological research: A prospectus. Aquaculture. 2006;259: 444–448.

85. Ferse SCA, Kunzmann A. Effects of Concrete-Bamboo Cages on Coral Fragments: Evaluation of a Low-Tech Method Used in Artisanal Ocean-Based Coral Farming. Journal of Applied Aquaculture. Taylor & Francis Group; 2009;21: 31–49. doi:10.1080/10454430802694538

86. Carne L, Kaufman L, Scavo K. Measuring success for Caribbean acroporid restoration: key results from ten years of work in southern Belize. 2016.

87. Calle-Triviño J, Cortés-Useche C, Sellares-Blasco RI, Arias-González JE. Assisted fertilization of threatened Staghorn Coral to complement the restoration of nurseries in Southeastern Dominican Republic. Regional Studies in Marine Science. Elsevier; 2018;18: 129–134. doi:10.1016/j.rsma.2018.02.002

88. Linden B, Rinkevich B. Creating stocks of young colonies from brooding coral larvae, amenable to active reef restoration. Journal of Experimental Marine Biology and Ecology. Elsevier; 2011;398: 40–46. doi:10.1016/j.jembe.2010.12.002

89. National Marine Fisheries Service. Recovery Plan for Elkhorn (Acropora palmata) and Staghorn (A. cervicornis) Corals

. Silver Spring, MD; Mar, 2015 p. 167.

90. Carne L, Kaufman L, Scavo K. Measuring success for Caribbean acroporid restoration: key results from ten years of work in southern Belize.

91. Ross AM. Genet and reef position effects in out-planting of nursery-grown Acropora cervicornis (Scleractinia: Acroporidae) in Montego Bay, Jamaica. Revista de Biología Tropical. http://creativecommons. org/licenses/by/3.0; 2014;62: 318–329.

92. Bowden-Kerby A. Restoration of the threatenedAcropora cervicorniscorals: intraspecific variation as a factor in mortality, growth, and self-attachment. Ft. Lauderdale, Florida, USA; 2008. pp. 1194–1198.

93. Goergen EA, Gilliam DS. Outplanting technique, host genotype, and site affect the initial success of outplanted *Acropora cervicornis*. PeerJ. 2018;6: e4433. doi:10.7717/peerj.4433

94. Baums IB. A restoration genetics guide for coral reef conservation. Molecular Ecology. 2008;17: 2796–2811.

95. Ladd MC, Miller MW, Hunt JH, Sharp WC, Burkepile DE. Harnessing ecological processes to facilitate coral restoration. Frontiers in Ecology and the Environment. John Wiley & Sons, Ltd; 2018;16: 239–247. doi:10.1002/fee.1792

96. Page CA, Vaughan D. The cultivation of massive corals using “micro-fragmentaion” for the ‘reskinning’ of degraded coral reefs. Benthic Ecology Meeting.

97. Forsman ZH, Page CA, Toonen RJ, Vaughan D. Growing coral larger and faster: micro-colony-fusion as a strategy for accelerating coral cover. PeerJ. PeerJ Inc; 2015;3: e1313. doi:10.7717/peerj.1313

98. Hall ER, DeGroot BC, Fine M. Lesion recovery of two scleractinian corals under low pH conditions: Implications for restoration efforts. Mar Pollut Bull. Elsevier; 2015;100: 321–326.

99. Harrison PL, Wallace CC, Dubinsky Z. Reproduction, dispersal and recruitment of scleractinian corals. Ecosystems of the world coral reefs. Reproduction, dispersal and recruitment of scleractinian corals. Elsevier, Amnsterdam; 1990.

100. Richmond RH. Coral-reefs - present problems and future concerns resulting from anthropogenic disturbance. Am Zool. 1993;33: 524–536.

101. Jones GP, Almany GR, Russ GR, Sale PF, Steneck RS, Van Oppen M, et al. Larval retention and connectivity among populations of corals and reef fishes: history, advances and challenges. Coral Reefs. Springer; 2009;28: 307–325.

102. van Oppen MJH, Gates RD, Blackall LL, Cantin NE, Chakravarti LJ, Chan WY, et al. Shifting paradigms in restoration of the world's coral reefs. Glob Chang Biol. 2017;23: 3437–3448.

103. Omori M. Success of mass culture of Acropora corals from egg to colony in open water. Coral Reefs. Springer-Verlag; 2005;24: 563–563. doi:10.1007/s00338-005-0030-4

104. Omori M, Iwao K, Tamura M. Growth of transplanted Acropora tenuis 2 years after egg culture. Coral Reefs. Springer-Verlag; 2007;27: 165–165. doi:10.1007/s00338-007-0312-0

105. IWAO K, Omori M, Taniguchi H, TAMURA M. Transplanted Acropora tenuis (Dana) spawned first in their life 4 years after culture from eggs. Galaxea, Journal of Coral Reef Studies. The Japanese Coral Reef Society; 2010;12: 47–47.

106. Raymundo LJ, Maypa AP. Getting bigger faster: mediation of size-specific mortality via fusion in juvenile coral transplants. Ecol Appl. 2004;14: 281–295.

107. Villanueva RD, Baria MVB, Cruz dela DW. Growth and survivorship of juvenile corals outplanted to degraded reef areas in Bolinao-Anda Reef Complex, Philippines. Marine Biology Research. Taylor \& Francis; 2012;8: 877–884.

108. Baria MVB, Cruz dela DW, Villanueva RD, Guest JR. Spawning of Three-Year-Old Acropora Millepora Corals Reared from Larvae in Northwestern Philippines. B Mar Sci. University of Miami - Rosenstiel School of Marine and Atmospheric Science; 2012;88: 61–62. doi:10.5343/bms.2011.1075

109. Guest JR, Baria MV, Gomez ED, Heyward AJ, Edwards AJ. Closing the circle: is it feasible to rehabilitate reefs with sexually propagated corals? Coral Reefs. Springer; 2014;33: 45–55.

110. Chamberland VF, Petersen D, Guest JR, Petersen U, Brittsan M, Vermeij MJA. New Seeding Approach Reduces Costs and Time to Outplant Sexually Propagated Corals for Reef Restoration. Sci Rep. nature.com; 2017;7: 18076.

111. Edwards AJ, Guest JR, Heyward AJ, Villanueva RD, Baria MV, Bollozos ISF, et al. Direct seeding of mass-cultured coral larvae is not an effective option for reef rehabilitation. Mar Ecol Prog Ser. 2015;525: 105–116. doi:10.3354/meps11171

112. Heyward AJ, Smith LD, Rees M, Field SN. Enhancement of coral recruitment by in situ mass culture of coral larvae. Mar Ecol Prog Ser. 2002;230: 113–118. doi:10.2307/24865098

113. Suzuki G, Arakaki S, Suzuki K, Iehisa Y, Hayashibara T. What is the optimal density of larval seeding in Acropora corals? Fisheries Science. Springer; 2012;78: 801–808.

114. Harrison P, Villanueva R, la Cruz de D. Coral Reef Restoration Using Mass Coral Larval Reseeding. Australian Centre for International Agricultural Research, Canberra, Australia. 2016.

115. Burt J, Bartholomew A, Bauman A, Saif A, Sale PF. Coral recruitment and early benthic community development on several materials used in the construction of artificial reefs and breakwaters. J Exp Mar Bio Ecol. 2009;373: 72–78.

116. Baine M. Artificial reefs: a review of their design, application, management and performance. Ocean Coast Manage. 2001;44: 241–259.

117. Burt J, Bartholomew A, Bauman A, Saif A, Sale PF. Coral recruitment and early benthic community development on several materials used in the construction of artificial reefs and breakwaters. Journal of Experimental Marine Biology and Ecology. 2009;373: 72–78. doi:10.1016/j.jembe.2009.03.009

118. Thanner SE, McIntosh RJ, Blair SM. Development of benthic and fish assemblages on artificial reef materials compared to adjacent natural reef assemblages in Miami-Dade County, Florida. B Mar Sci. 2006;78: 57–70.

119. Hannes AR, Floyd LS. Coral recruitment and community development: the Broward County artificial reef compared to adjacent hardbottom areas, five years post-deployment. Ft. Lauderdale, FL, USA; 2008. pp. 1245–1248.

120. Williams SL, Sur C, Janetski N, Hollarsmith JA, Rapi S, Barron L, et al. Large-scale coral reef rehabilitation after blast fishing in Indonesia. Restoration Ecology. Wiley Online Library; 2019;27: 447–456.

121. Zepeda-Centeno C, Mariño-Tapia I, Mcleod E, Rodrigues-Martínez R, Alvarez-Filip L, Banaszak AT, et al. Guidance Document for Reef Management and Restoration to Improve Coastal Protection: Recommendations for Global Applica- tions based on lessons learned in Mexico. The Nature Conservancy, Mexico; p. 81.

122. Reguero BG, Beck MW, Agostini VN, Kramer P, Hancock B. Coral reefs for coastal protection: A new methodological approach and engineering case study in Grenada. Journal of Environmental Management. Academic Press; 2018;210: 146–161. doi:10.1016/j.jenvman.2018.01.024

123. Perkol-Finkel S, Benayahu Y. Community structure of stony and soft corals on vertical unplanned artificial reefs in Eilat (Red Sea): comparison to natural reefs. Coral Reefs. 2004;23: 195–205.

124. Thanner SE, McIntosh RJ, Blair SM. Development of benthic and fish assemblages on artificial reef materials compared to adjacent natural reef assemblages in Miami-Dade County, Florida. Bull Mar Sci. 2006;78: 57–70.

125. Blakeway D, Byers M, Stoddart J, Rossendell J. Coral colonisation of an artificial reef in a turbid nearshore environment, Dampier Harbour, western Australia. Harder T, editor. PLoS ONE. Public Library of Science; 2013;8: e75281. doi:10.1371/journal.pone.0075281

126. Jaap WC. Coral reef restoration. Ecological Engineering. 2000;15: 345–364.

127. Fox HE, MOUS PJ, PET JS, MULJADI AH, CALDWELL RL. Experimental Assessment of Coral Reef Rehabilitation Following Blast Fishing. Conserv Biol. Blackwell Publishing Inc; 2005;19: 98–107. doi:10.1111/j.1523-1739.2005.00261.x

128. Hudson JH, Diaz R. Damage survey and restoration of M/V Wellwood grounding site, Molasses Reef, Key Largo National Marine Sanctuary, Florida. Townsville; 1988. pp. 231–236. Available: http://www.aoml.noaa.gov/general/lib/CREWS/mlrf_25.pdf

129. Goreau TJ, Hilbertz W. Reef restoration using sea water electrolysis in Jamaica. Panama; 1996.

130. Hilbertz W. Electrodeposition of minerals in sea water: Experiments and applications. IEEE Journal of Oceanic Engineering. IEEE; 1979;4: 94–113.

131. Goreau TJ, Hilbertz W. Reef restoration using sea water electrolysis in Jamaica. Panama; 1996. p. 75.

132. Goreau TJ. Electrical Stimulation Greatly Increases Settlement, Growth, Survival, and Stress Resistance of Marine Organisms. Natural Resources. 2014;5: 527–537.

133. Sabater MG, Yap HT. Growth and survival of coral transplants with and without electrochemical deposition of CaCo3. Journal of Experimental Marine Biology and Ecology. 2002;272: 131–146. doi:10.1016/S0022-0981(02)00051-5

134. Van Treeck P, Schuhmacher H. Artificial Reefs Created by Electrolysis and Coral Transplantation: An Approach Ensuring the Compatibility of Environmental Protection and Diving Tourism. Estuar Coast Shelf S. Academic Press; 1999;49: 75–81. doi:10.1016/S0272-7714(99)80011-0

135. Schuhmacher H, Van Treeck P, Eisinger M, Paster M. Transplantation of coral fragments from ship groundings on electrochemically formed reef structures. 2002. pp. 983–990.

136. Sabater MG, Yap HT. Growth and survival of coral transplants with and without electrochemical deposition of CaCO3. Journal of Experimental Marine Biology and Ecology. 2002;272: 131–146.

137. Sabater MG, Yap HT. Long-term effects of induced mineral accretion on growth, survival and corallite properties of *Porites cylindrica* Dana. J Exp Mar Bio Ecol. 2004;311: 355–374.

138. Eisinger M. Beiträge zu ökologischen und ökonomischen Aspekten der Korallentransplantation auf elektrochemisch erzeugte Substrate als Methode zur Rehabilitation degradierter Korallenriffe. Department Biology and Geography. University of Duisburg-Essen; 2005.

139. Romatzki SBC. Influence of electrical fields on the performance of Acropora coral transplants on two different designs of structures. Mar Biol Res. Taylor \& Francis; 2014;10: 449–459.

140. Borell EM, Romatzki SBC, Ferse SCA. Differential physiological responses of two congeneric scleractinian corals to mineral accretion and an electric field. Coral Reefs. 2010;29: 191–200.

141. Goreau TJ. Electrical Stimulation Greatly Increases Settlement, Growth, Survival, and Stress Resistance of Marine Organisms. Natural Resources. Scientific Research Publishing; 2014;5: 527–537. doi:10.4236/nr.2014.510048
