# Supplementary material for: Coral restoration – A systematic review of current methods, successes, failures and future directions
Source: PLoS One. 2020 Jan 30;15(1):e0226631. doi: 10.1371/journal.pone.0226631 (PMC6992220; doi:10.1371/journal.pone.0226631)
Supplement: S1 Fig — (DOC) [file pone.0226631.s005.doc]

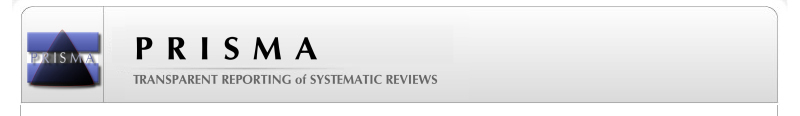
**PRISMA 2009 Flow Diagram**

**Screening**

**Included**

**Eligibility**

**Identification**

Records identified through database searching
(n = 738 )

Additional records identified through other sources
(n = 78 )

Records after duplicates removed
(n = 816 )

Records screened
(n = 816 )

Records excluded
(n = 595 )

Full-text articles assessed for eligibility
(n = 335 )

Full-text articles excluded, with reasons
(n = 110 )

Studies included in qualitative synthesis
(n = 362 )

Studies included in quantitative synthesis (meta-analysis)
(n = 0 )
