# Supplementary material for: Coral restoration – A systematic review of current methods, successes, failures and future directions
Source: PLoS One. 2020 Jan 30;15(1):e0226631. doi: 10.1371/journal.pone.0226631 (PMC6992220; doi:10.1371/journal.pone.0226631)
Supplement: Attachment — Submitted filename: Detailed responses to reviewer comments.pdf [file pone.0226631.s006.pdf]

Detailed responses to reviewer comments:

Reviewer 1:

1. *Some minor comments and suggestions are provided in the annotated pdf file.*
   Author response: All comments provided in PDF have been addressed.

2. *I know that this review is focused on corals but I think that a general comment, at the end of the ms, briefly discussing the current situation in restoring other marine habitats could be useful to generalize the overall difficulty of these procedures. For instance, also in the seagrass restoration practices the same issues have raised, linked to the short period of projects/monitoring activities and to the small areas covered by these projects.*
   Author response: This is an excellent suggestion by the reviewer. We have expanded the existing section discussing this, to more clearly provide comparisons with restoration in other ecosystems. **[L470-476]**

3. *As a final suggestion, Authors often underlined the lack of standardization in methods, materials, metrics, protocols, etc. Being this review the first one produced on this topic to date, I think it will become a reference in the future research on coral restoration. Thus, the Authors could venture to propose a possible first protocol for coral restoration at the end of this review (maybe as future directions?), based on the great experience they gained through the critical analysis of lot of papers and reports. I'm sure they could suggest a set of techniques, materials, metrics, surfaces involved, monitoring period (depending on local conditions, cost, availability of materials and appropriateness based on stated objectives, as the Authors already recognized in this review) that showed to be the most adopted worldwide and the most effective. It could be the first step towards achieving standardization in coral restoration*.
   Author response: This is something we, as a research group are working on. However, we judged it would not be possible to provide a comprehensive best practice guide to monitoring within this review, as it would make the review a very bloated article. Instead, we aim to publish this guide as a standalone publication, in the coming year, potentially as part of a special issue in PlosONE (currently in negotiation/planning stage with the journal). Instead of a full blown best-practice monitoring guides, we instead opted for a section outlining some of the characteristics required in monitoring guidelines, and some of the common issues (in section titled "Objectives and monitoring").

Reviewer 2:

1. *While the authors provide an interesting database, data analysis essentially reduces to some summary statistics of the outcomes of the revewed restoration projects, without traditional meta-analysis comparisons. For example, studies are compared by using "average" survival measures of corals, without weighting for the different sample sizes and standard errors and without providing pooled measures of uncertainty. On the one side, I acknowledge that meta-analysis methods might be prohibitive in this framework, due to the extreme heterogenety of the sources that*

have been collected by the authors. On the other side, the lack of statistical comparisons reduces this paper to a qualitative review.
Author response: Due to the wide scope of this review, merging information from widely varied sources, the collected data is heterogeneous and poorly suited to traditional statistics used in meta-analysis. Taking the editor's suggestion, we have therefore amended the text to more clearly define the review as a qualitative rather than quantitative review, to reduce statistical ambiguity. The following text has been added to make this clear to the reader:

[L273-276]: Due to the high heterogeneity of information available from such a diverse range of sources, we were precluded from performing quantitative statistical meta-analyses. Instead, this is a qualitative review using summary statistics to evaluate and contrast different restoration method outcomes.
